# Pathological structural conversion of α-synuclein at the mitochondria induces neuronal toxicity

**DOI:** 10.1038/s41593-022-01140-3

**Published:** 2022-08-30

**Authors:** Minee L. Choi, Alexandre Chappard, Bhanu P. Singh, Catherine Maclachlan, Margarida Rodrigues, Evgeniya I. Fedotova, Alexey V. Berezhnov, Suman De, Christopher J. Peddie, Dilan Athauda, Gurvir S. Virdi, Weijia Zhang, James R. Evans, Anna I. Wernick, Zeinab Shadman Zanjani, Plamena R. Angelova, Noemi Esteras, Andrey Y. Vinokurov, Katie Morris, Kiani Jeacock, Laura Tosatto, Daniel Little, Paul Gissen, David J. Clarke, Tilo Kunath, Lucy Collinson, David Klenerman, Andrey Y. Abramov, Mathew H. Horrocks, Sonia Gandhi

**Affiliations:** 1 Department of Clinical and Movement Neurosciences, UCL Queen Square Institute of Neurology, London, UK; 2 The Francis Crick Institute, London, UK; 3 Aligning Science Across Parkinson’s (ASAP) Collaborative Research Network, Chevy Chase, MD USA; 4 EaStCHEM School of Chemistry, University of Edinburgh, Edinburgh, UK; 5 School of Physics, University of Edinburgh, Edinburgh, UK; 6 Department of Chemistry, University of Cambridge, Cambridge, UK; 7 Dementia Research institute at University of Cambridge, Cambridge, UK; 8 Institute of Cell Biophysics, Russian Academy of Sciences, Pushchino, Russia; 9 Cell Physiology and Pathology Laboratory, Orel State University, Orel, Russia; 10 Istituto di Biofisica, National Council of Research, Trento, Italy; 11 MRC Laboratory for Molecular Cell Biology, University College London, London, UK; 12 NIHR Great Ormond Street Hospital Biomedical Research Centre, London, UK; 13 Centre for Regenerative Medicine, Institute for Stem Cell Research, School of Biological Sciences, University of Edinburgh, Edinburgh, UK

**Keywords:** Parkinson's disease, Biophysics, Mitochondria

## Abstract

Aggregation of alpha-synuclein (α-Syn) drives Parkinson’s disease (PD), although the initial stages of self-assembly and structural conversion have not been directly observed inside neurons. In this study, we tracked the intracellular conformational states of α-Syn using a single-molecule Förster resonance energy transfer (smFRET) biosensor, and we show here that α-Syn converts from a monomeric state into two distinct oligomeric states in neurons in a concentration-dependent and sequence-specific manner. Three-dimensional FRET-correlative light and electron microscopy (FRET-CLEM) revealed that intracellular seeding events occur preferentially on membrane surfaces, especially at mitochondrial membranes. The mitochondrial lipid cardiolipin triggers rapid oligomerization of A53T α-Syn, and cardiolipin is sequestered within aggregating lipid–protein complexes. Mitochondrial aggregates impair complex I activity and increase mitochondrial reactive oxygen species (ROS) generation, which accelerates the oligomerization of A53T α-Syn and causes permeabilization of mitochondrial membranes and cell death. These processes were also observed in induced pluripotent stem cell (iPSC)–derived neurons harboring A53T mutations from patients with PD. Our study highlights a mechanism of de novo α-Syn oligomerization at mitochondrial membranes and subsequent neuronal toxicity.

## Abstract (web summary)

This study tracked the initial self-assembly, oligomerization and structural conversion of α-synuclein inside neurons. Early seeding events occur on mitochondrial membranes, where oligomerization induces mitochondrial dysfunction and neuronal loss.

## Main

The presence of aggregates rich in α-Syn is a hallmark of synucleinopathies, a subset of neurodegenerative diseases that encompasses PD, dementia with Lewy bodies (DLB) and multiple system atrophy (MSA)^1–5^. The α-Syn protein plays a central role in the pathogenesis of these diseases. Genetic studies have confirmed that mutations and multiplications of the gene *SNCA* (https://www.ncbi.nlm.nih.gov/gene/6622) on chromosome 4q21–23, encoding α-Syn, cause early-onset familial PD^1,6–8^, with widespread deposition of α-Syn aggregates in the brain. α-Syn is an intrinsically disordered protein and, during pathology, undergoes a conformational change to β-sheet-rich structures including oligomers, protofibrils and insoluble fibrils that finally accumulate in Lewy bodies (LBs) (reviewed in ref. ^9^). Although it is the insoluble end‐stage species of protein aggregation that has traditionally defined disease, it is the soluble intermediate oligomers that are markedly toxic, inducing aberrant calcium signaling, generating ROS, mitochondrial dysfunction and neuronal cell death^2,10–13^. Early dynamic processes in the formation of α-Syn oligomers are difficult to investigate due to their intrinsically transient, heterogeneous nature and low abundance (reviewed in ref. ^14^). To overcome this, single-molecule fluorescence methods have been developed to observe protein interactions and conformations, which broadly rely on the detection of transfer of excitation energy between two fluorophores when sufficiently close (reviewed in ref. ^15^). Employing smFRET, we previously tracked α-Syn aggregation in vitro, distinguished different structural groups of oligomers formed during aggregation and compared the kinetics of oligomer formation of SNCA mutations, highlighting the increased propensity for A53T α-Syn to form cytotoxic oligomers^16,17^. However, it has not yet been possible to capture and characterize the early dynamic process of aggregation inside the native human environment and determine its effects on cellular homeostasis.

In this study, we integrated sensitive biophysical approaches—a FRET biosensor, smFRET and three-dimensional (3D) FRET-CLEM—to precisely track the kinetics of α-Syn aggregation and to visualize the early oligomerization events inside neurons. Here we show how early seeding events on lipid membranes trigger aggregation, and we reveal a mechanism by which mutant α-Syn aggregates in mitochondria and alters mitochondrial function, which leads ultimately to toxicity.

## Results

### Measuring oligomerization using a FRET biosensor

**Fig. 1:**
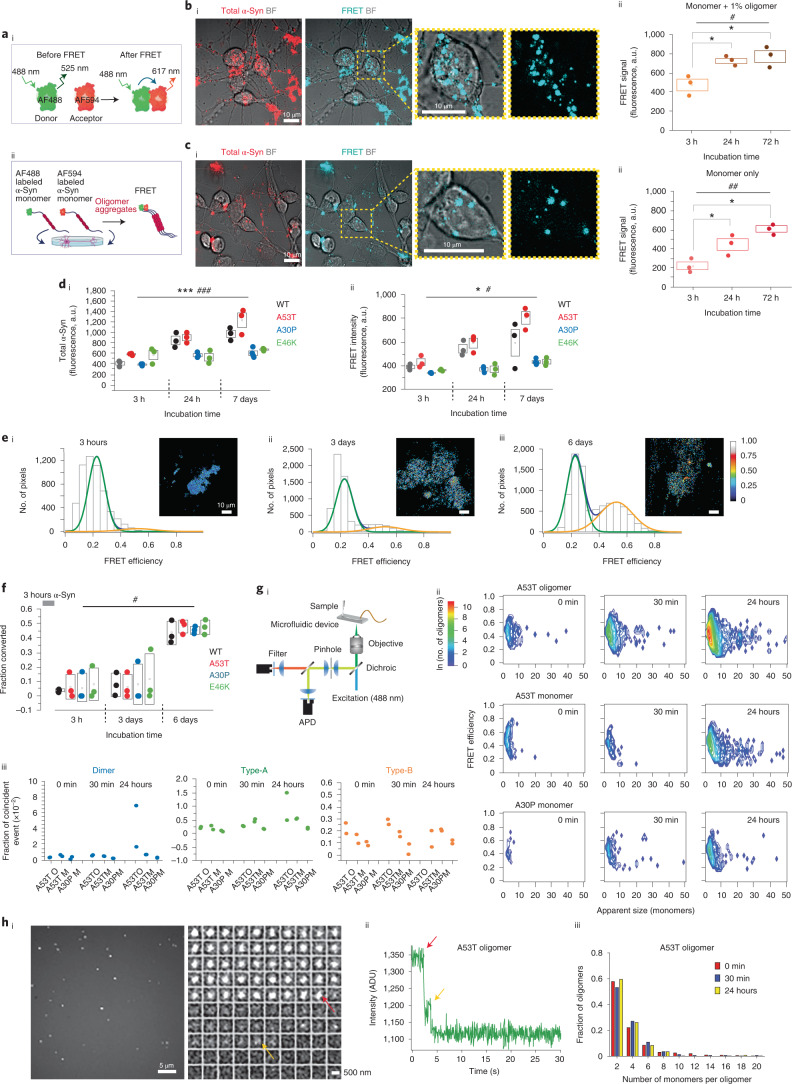
FRET sensor detects rapid intracellular oligomerization of A53T α-Syn. **ai**, Schematic illustration showing how FRET sensor detects aggregation. **aii**, AF488-α-Syn and AF594-α-Syn monomers are applied to cells, and the FRET signal is detected. **bi**, Representative bright-field (BF) FRET images after 72-hour incubation with oligomers. **bii**, Application of 500 nM WT oligomeric α-Syn exhibits detectable FRET, which increases over time (*n* = 3 independent experiments). **ci**, Representative FRET images after 72-hour incubation with monomers. **cii**, Application of 500 nM monomeric α-Syn exhibits low FRET signal initially, followed by an increase in FRET over time (*n* = 3 independent experiments). **di**,**dii**, A53T monomer exhibits the highest intracellular accumulation of α-Syn and the highest intracellular FRET intensity over time (*n* = 3 or 4 independent experiments). **ei**–**eiii**, FRET efficiency was calculated and binned into histograms that were fit to two Gaussian distributions. After 3 hours, only a low-FRET-efficiency population (centered at *E* = 0.24) was present. After 3 days, a second higher-FRET-efficiency population appeared (*E* = 0.48), and the fraction of this increased over time. **f**, Fraction of the high-FRET-efficiency population (out of total FRET events) increases over time for the WT and all mutants. Fitting error is shown in Extended Data Fig. 2c (*n* = 3 or 4 independent experiments). **gi**, Single-molecule confocal microscopy under conditions of fast flow used to analyze cell lysates. **gii**, 2D contour plots of approximate oligomer size and FRET efficiency after application of the monomers and oligomers. Both the number of events and the size of the oligomers increase over time in all cases. **giii**, Number and type of oligomeric events in cell lysates from the monomer/oligomer-treated cells. Data are represented as data ± s.d., as fraction of coincident events (*n* = 2 independent samples). Fitting error is shown in Extended Data Fig. 3a. **hi**, Photobleaching step analysis for A53T-oligomer-treated cells. **hii**, Step-fit example of a single A53T oligomer (24 hours) intensity trace (intensity is plotted as analogue-to-digital units (ADU)). **hiii**, Each step indicates photobleaching of a single fluorophore, from which oligomer size can be estimated. Note: Data are represented as data ± s.e.m. (box) unless otherwise mentioned. Detailed statistical information is shown in Supplementary Table 1. See also Extended Data Figs. 1–4. a.u., arbitrary units. Source data

**Extended Data Fig. 1:**
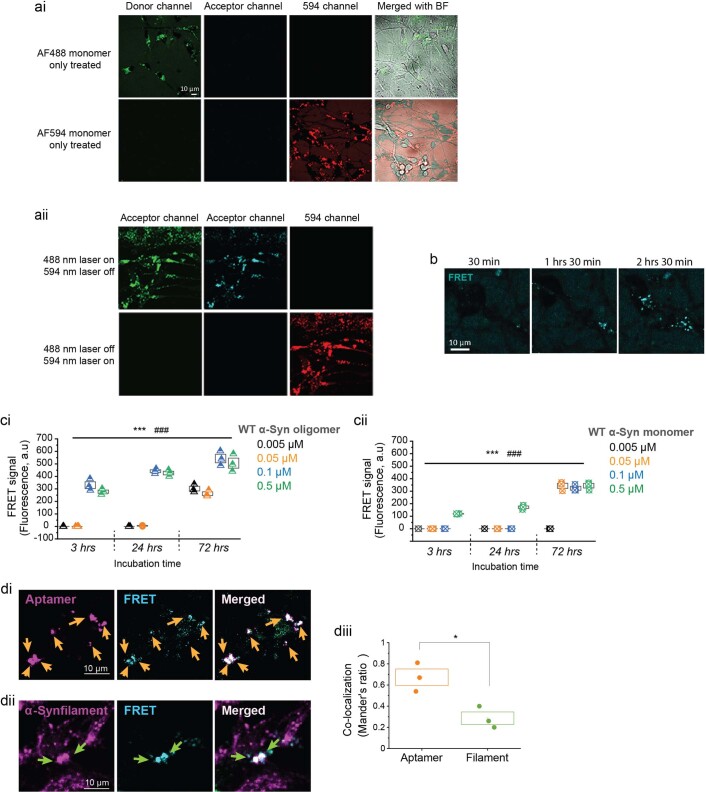
Related to main Fig. 1. **(ai)** α-Syn-AF488-monomer or α-Syn-AF594-monomer alone did not induce a FRET signal. **(aii)** FRET was not induced by 594 nm excitation. **(b)** Time-lapse representative images of FRET showing intracellular localization of α-Syn assemblies after adding AF488-α-Syn and AF-594-α-Syn monomers to the media. **(ci & ii)** Intracellular FRET signal induced by WT α-Syn (ci; oligomer, cii; monomer) occurred in a concentration- and time-dependent manner (n = 3 independent experiments). The minimum concentration to induce FRET within 72 hours was 5 nM oligomer; the minimum monomer concentration to induce FRET at 72 hours was 50 nM, whilst 500 nM monomer induced a FRET signal within 3 hours **(di & ii)** FRET signal colocalized with an amyloid oligomer-specific aptamer and partially with an α-Syn filament antibody. (**diii)** Co-localization was quantified using Mander’s ratio (n = 3 independent experiments). *Note*. Data are represented as Data ± SEM (box). *#*p* < 0.05, **##*p* < 0.005, ***###*p* < 0.0005. Detailed statistical information is provided in Supplementary Table 1. Source data

For visualization of α-Syn, fluorescent labels were introduced at an alanine-to-cysteine mutation at residue 90 (A90C), which is located at the periphery of the structure proposed to be highly organized in the fibrillar form and has negligible effects on both the aggregation and toxicity of α-Syn^10,18^. Two populations of fluorescently tagged α-Syn, one labeled with Alexa Fluor 488 (α-Syn-AF488) and the second with Alexa Fluor 594 (α-Syn-AF594) (Fig. 1ai,aii), were mixed and added to neurons, and the intracellular accumulation of α-Syn was visualized by measuring the intensity of AF594 α-Syn within the cells using direct excitation with 594-nm irradiation (Fig. 1bi,ci and Extended Data Fig. 1ai,aii). This direct excitation signal gives a measure of the total α-Syn, regardless of its aggregation state. The formation of oligomers was visualized via the presence of signal from the acceptor fluorophore (AF549) after excitation of the donor fluorophore with 488-nm irradiation, which occurs as energy is non-radiatively transferred from AF488 to AF594. We refer to this as the FRET signal (Extended Data Fig. 1b), and it can only occur when the fluorophores are in close proximity (<10 nm), as is the case within aggregates.

We first applied 500 nM oligomeric α-Syn (containing ~1% oligomer and ~99% monomer) to primary neurons: uptake of α-Syn resulted in a direct excitation signal (total α-Syn) and a FRET signal (oligomer formation), which both increased in intensity over time, reflecting the continual uptake of α-Syn and subsequent aggregation inside cells (Fig. 1bi,bii).

The kinetics of aggregation are dependent on α-Syn concentration: application of a range of initial concentrations of 5–50 nM oligomeric α-Syn induced oligomerization in a time-dependent and concentration-dependent manner (Extended Data Fig. 1ci). To verify that the intracellular FRET signal is a biosensor of aggregate formation, we confirmed that the FRET signal co-localized with an ATTO425-labeled aptamer specific to β-sheet-rich aggregates (Extended Data Fig. 1di,diii)^19^ and with a conformation-specific antibody (Extended Data Fig. 1dii,diii)^12^.

**Extended Data Fig. 2:**
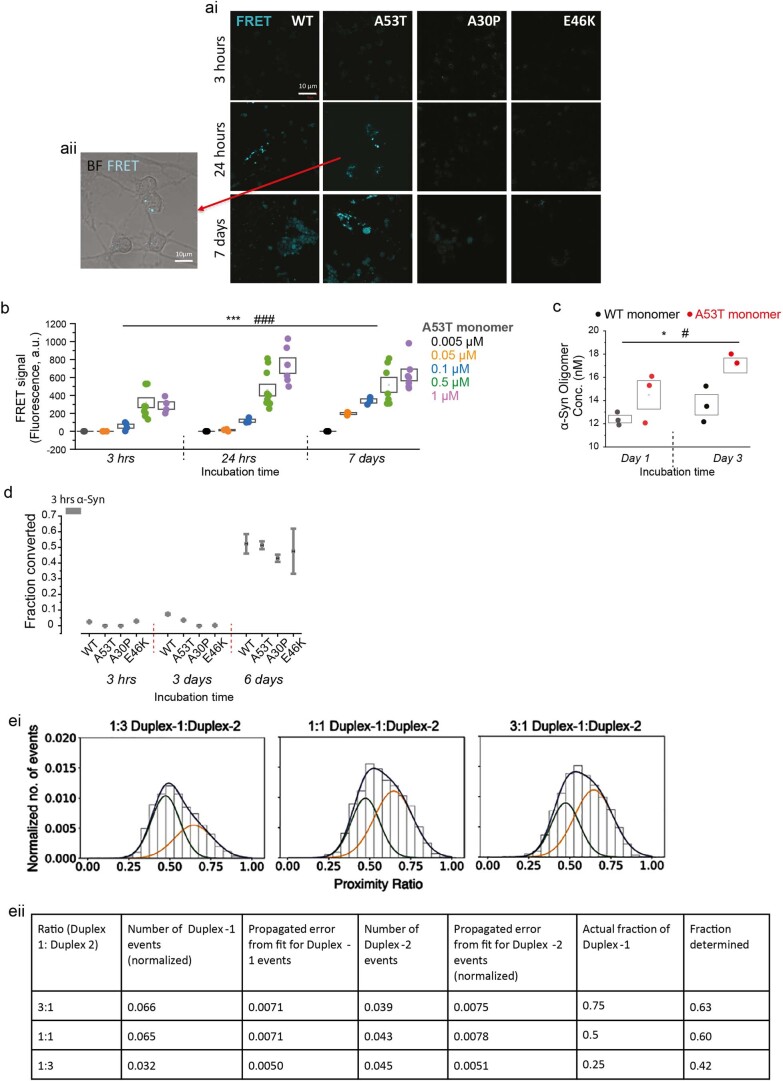
Related to main Fig. 1. (**ai**,**aii**) Representative brightfield (BF) and confocal images of FRET signal induced by application of WT or mutant α-Syn in main Fig. 1d. **(b)** Intracellular FRET signal induced by A53T α-Syn monomers occurred in a concentration- and time-dependent manner (n = 3, 6 or 10 fields imaged over 2 or 4 independent experiments; more experiments were performed with the concentrations that produced higher FRET signal). **(c)** Higher oligomer concentration was measured in A53T monomer-treated cells compared to WT-treated cells after 3 days of incubation. Concentration was measured using a Human Synuclein, alpha (non A4 component of amyloid precursor) oligomer (SNCA oligomer) ELISA kit (CSB-E18033h, Generon) (n = 3 independent experiments). **(d)** fitting error for main Fig. 1f. Data are represented as mean ± SD. **(ei)** FRET histograms of different ratios of duplexes analyzed using single-molecule confocal microscopy. **(eii)** Number and the fraction of detected species for different mixtures of Duplex-1 and Duplex-2. *Note*. Data are represented as Data ± SEM (box). *#*p* < 0.05, **##*p* < 0.005, ***###*p* < 0.0005. Detailed statistical information is provided in Supplementary Table 1. Source data

Next, we tested whether the application of α-Syn monomers alone (in the absence of oligomers) resulted in self-assembly and oligomer formation. After an initial lag phase, the FRET signal intensity increased (Fig. 1ci,cii). Oligomerization of the monomer occurred in a time-dependent and concentration-dependent manner (Extended Data Fig. 1cii). Within 3 hours, 500 nM monomer induced a FRET signal (oligomer formation), whereas 50 nM induced a FRET signal by 48 hours. We treated cells with 500 nM A53T, A30P, E46K or wild-type (WT) α-Syn monomers and measured both the total α-Syn and oligomer formation at different timepoints. Total α-Syn uptake and the FRET signal increased for WT and all mutants, showing a time-dependent increase in aggregate formation (Fig. 1di,dii and Extended Data Fig. 2ai,aii). Of note, A53T showed the greatest increase in both direct excitation signal and intracellular FRET signal over time, indicating increased uptake and oligomerization compared to the other mutations (Fig. 1di,dii). Oligomerization is concentration dependent for A53T α-Syn (Extended Data Fig. 2b). The formation of aggregates was further confirmed using an α-Syn oligomer-specific ELISA (Extended Data Fig. 2c).

### Structural conversion measured by FRET efficiency

smFRET measurements can identify the in vitro structural conversion from less toxic, loosely associated ‘Type-A’ oligomers into toxic, proteinase-K-resistant, β-sheet-rich ‘Type-B’ oligomers^18^. Taking advantage of the intracellular FRET signal detected here, we determined whether distinct FRET populations of oligomers could form within neurons by calculating the FRET efficiency (E) of the aggregates within the cells (Equation 1 and Methods).

FRET efficiency histograms were generated from the intracellular aggregates after a short 3-hour incubation with WT 500 nM α-Syn monomer. The histograms were globally fit to two Gaussian distributions and integrated to obtain the number of converted oligomers in each sample (Fig. 1ei–eiii). The FRET efficiency increased over time for WT α-Syn, showing that the protein can oligomerize and structurally convert inside cells. At early timepoints (3 hours and 3 days), only the population of low-FRET-efficiency (centered at *E* = 0.24) oligomers were present. After 3 days, a second population of higher-FRET-efficiency oligomers (centered at *E* = 0.48) appeared. Assembly from monomer to oligomer occurred rapidly with a short lag phase (<3 hours), whereas conversion to a high-FRET oligomer occurred over days. Short incubation with A53T, A30P and E46K mutants (Fig. 1f and Extended Data Fig. 2d) revealed a similar structural conversion in cells.

### A53T shows accelerated oligomerization and reduced lag phase

**Extended Data Fig. 3:**
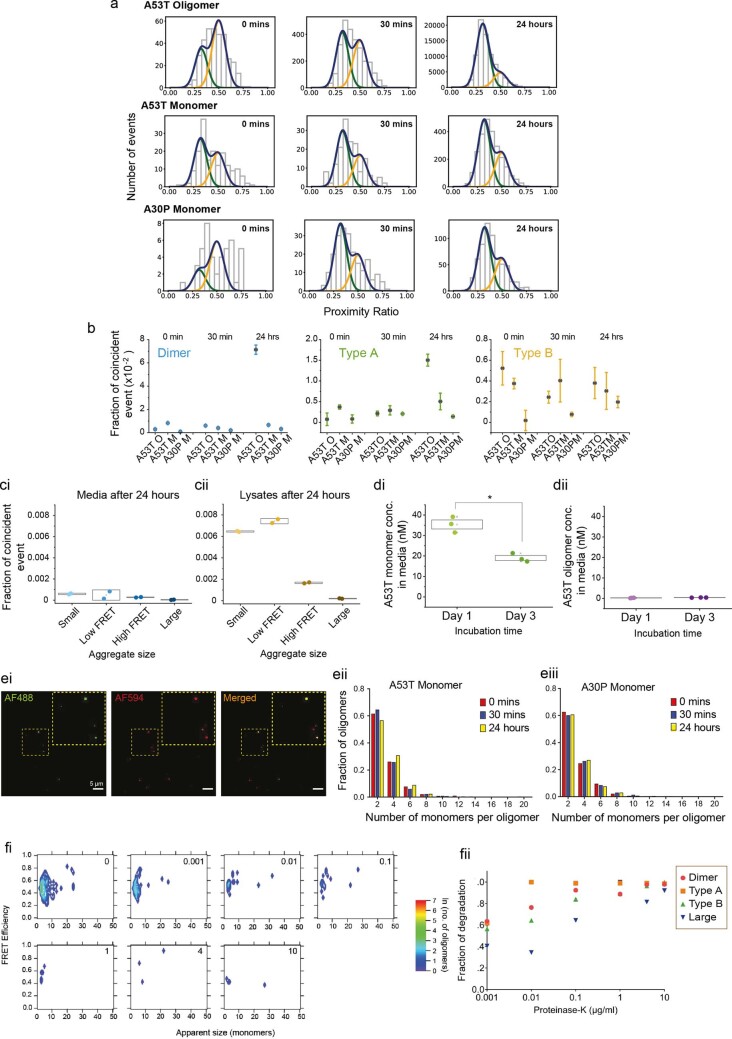
Related to main Fig. 1. **(a)** smFRET efficiency histograms of cell lysate data from Fig. 1g. Oligomer populations were fit (using a custom script written in Igor Pro, Wavemetrics) for medium-sized oligomers (deemed to be between 3 and 20 subunits). Oligomers were determined to be either low FRET (green fit line) or high FRET (orange) based on their histogram population distributions. At 24 hours, the number of oligomers detected was highest for cells treated with A53T oligomers, followed by cells treated with A53T monomers, with the cells treated with A30P monomers being the lowest in oligomer number. **(b)** Fitting error for main Fig. 1giii. Data expressed as mean ± SD. **(ci & ii)** smFRET shows there is a minimal number of oligomers (as a fraction of total α-Syn protein) in the media formed (di) and that oligomer formation is within cells (lysates) (dii) after the 24 hours incubation. Data are represented as a box plot showing replicate values and SD (n = 2 independent experiments). (**di & ii)** Comparison of A53T monomeric and oligomeric α-Syn from the media collected 1 day or 3 days after A53T monomer treatment (0.5 μM) using ELISA kits (Monomer; LEGEND MAX™ Human α-Synuclein ELISA Kit (SIG-38974, BioLegend), Oligomer; Human Synuclein, alpha (non A4 component of amyloid precursor) oligomer (SNCA oligomer) ELISA kit (CSB-E18033h, Generon). Over time, the monomeric α-Syn concentration in the media drops due to the intracellular uptake, whilst the oligomer concentration in the media constantly remained low (n = 3 independent experiments). **(ei)** TIRF images demonstrate that oligomers, but not fibrils, are detected in the lysates following 7 days of labeled A53T monomer treatment. **(eii & iii)** Photobleaching data demonstrating number of monomers in each oligomer detected in the lysate of cells treated with A53T monomer and A30P monomer respectively, in parallel with data shown in Fig. 1hiii. **(fi & ii)** Sensitivity of oligomers to Proteinase-K degradation *in vitro*. **(fi)** Two-dimensional contour plots of approximate oligomer sizes and FRET efficiency following application of increasing concentrations of proteinase-K to A53T aggregates formed at different timepoints. **(fii)** Fraction of degradation relative to the concentration of proteinase-K varies according to oligomer type. High-FRET aggregates (Type B) are more resistant to proteinase-K digestion than low-FRET (Type A). *Note*. Data are represented as Data ± SEM (box). *p < 0.05. Detailed statistical information is provided in Supplementary Table 1. Source data

To measure the FRET efficiencies of individual oligomers formed in cells, we used single-molecule confocal microscopy on cell lysates (Fig. 1gi)^18^. We detected both low-FRET-efficiency and high-FRET-efficiency oligomers with a range of different sizes as shown in the two-dimensional (2D) contour plots (Fig. 1gii). Fitting of the resultant FRET efficiency histograms (Extended Data Fig. 3a) allowed the small (dimeric), Type-A and Type-B oligomers to be quantified (Fig. 1giii; the fitting errors are shown in Extended Data Fig. 3b). To determine the systematic error in separating two different FRET populations using this approach, we analyzed mixtures of two different dual-labeled DNA duplexes that had different FRET efficiencies (see section in Methods and Extended Data Fig. 2ei,eii).

A53T-oligomer-treated cells (1% oligomer and 99% monomer) exhibited rapid assembly to form oligomeric species of increasing size over 24 hours. A53T-monomer-treated cells also exhibited rapid assembly into small oligomeric species at 0–30 minutes, which increased in size over 24 hours. Self-assembly and the early steps of aggregation were observed in A30P-treated cells, although less than A53T-treated cells (Fig. 1gii,giii and Extended Data Fig. 3a,b). We confirmed that the rapid assembly of oligomeric species is formed inside cells after intracellular uptake, as aggregate levels were negligible in the media and high in the cell lysates as measured by the fraction of coincident events (Extended Data Fig. 3ci,cii) and by ELISA (Extended Data Fig. 3di,dii).

We used total internal reflection fluorescence (TIRF) microscopy to image the oligomers (Fig. 1hi and Extended Data Fig. 3e). As each α-Syn monomer carries a single dye molecule, there is a stepwise decrease in intensity as each one photobleaches upon irradiation. By counting the number of these photobleaching steps, the number of monomers per oligomer can be determined (Fig. 1hi,hii) in lysates after the addition of A53T or A30P monomer and oligomer. Histograms showing the number of monomers per oligomer were generated (Fig. 1hiii and Extended Data Fig. 3ei–eiii). These size distributions (Fig. 1hiii) correlated well with those measured using single-molecule confocal microscopy; approximately half of the population of oligomers contained two monomers, whereas the rest contained 4–10 monomers. Due to simultaneous photobleaching of multiple fluorophores, larger oligomers could not be distinguished using this method.

Structural conversion from Type-A to Type-B oligomers is associated with increased resistance to proteinase K degradation. We tested the effect of increasing concentrations of proteinase K on A53T aggregates in vitro and showed that the Type-A oligomers formed at early timepoints are sensitive to low concentrations of proteinase K, whereas the Type-B and larger oligomers are more resistant to proteinase K degradation, requiring higher concentrations (Extended Data Fig. 3fi,fii).

**Extended Data Fig. 4:**
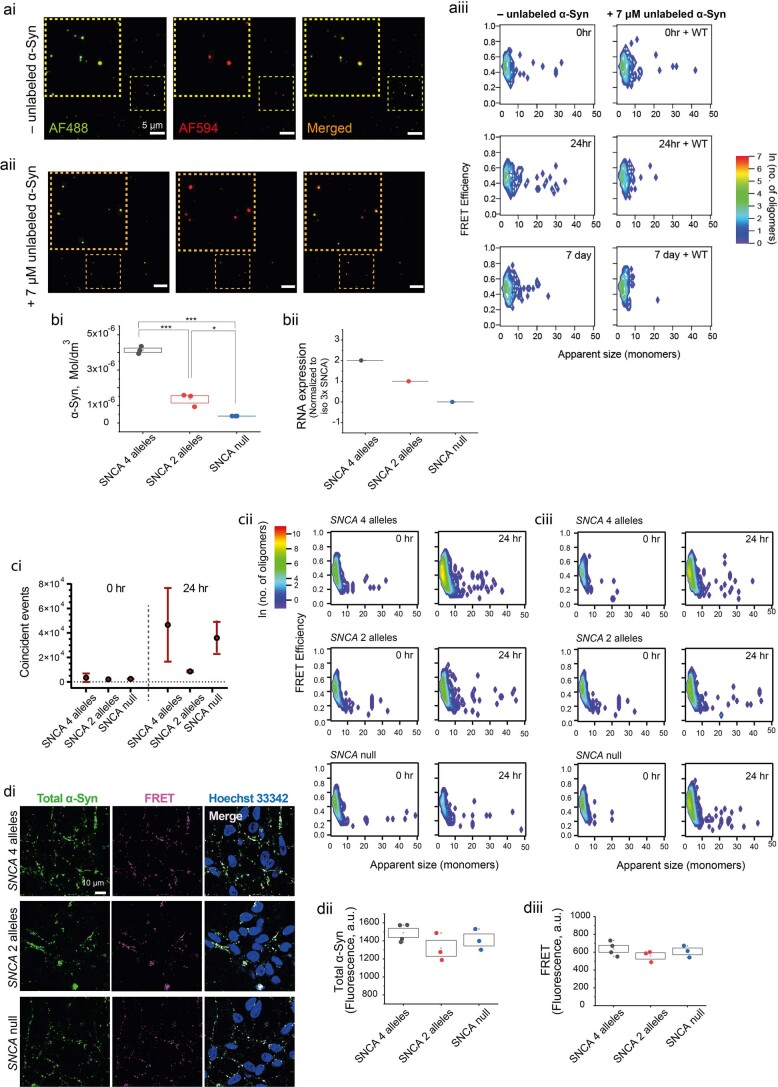
Related to main Fig. 1. **(a)** Formation of aggregates detected by FRET is not affected by unlabeled α-Syn. **(ai & ii)** Representative TIRF images of labeled recombinant A53T oligomers (formed after 7 days aggregation) that were formed in the absence (ai) and presence (aii) of 7 μM unlabeled wildtype (WT) α-Syn show that the presence of unlabeled protein has no effect on aggregate formation (n = 2 independent experiments) **(aiii)** Size and FRET distributions of oligomers detected using single-molecule confocal microscopy when labeled α-Syn aggregates in the absence (left) or presence (right) of 7 μM unlabeled WT α-Syn. **(b-d)** Concentrations of endogenous α-Syn did not affect aggregation kinetics measured by single-molecule (sm) FRET. **(bi & ii)** Allelic series of iPSC lines display different levels of α-Syn using Human Synuclein monomer ELISA kit (bi; n = 3 independent experiments), and *SNCA* mRNA by qPCR (bii; average of three wells, n = 1 independent experiment). **(c)** A53T-AF488 and AF-594 monomers (total 1 μM) were added to the three iPSC derived neurons and smFRET was performed on the cell lysates. **(ci)** Analysis of total FRET (coincident) events for the iPSC lines demonstrate non-significant higher mean events for *SNCA* 4 alleles at 24 hours, data shown as mean ± SD. **(cii & iii)** 2D contour plots from smFRET measurements of cell lysates show no difference in FRET efficiencies across the three cell lines, indicating aggregates formed in cells with different levels of the endogenous α-Syn show negligibe difference in FRET efficiency. (cii) and (ciii) show two separate replicates. **(di-iii)** FRET measurements obtained 24 hours after A53T-AF488 and AF594 monomer treatment show there is no difference in total α-Syn uptake (dii) and FRET intensity (diii) across the cells with different endogenous SNCA expression levels (n = 3 or 4 independent experiments). Experiments were performed at an early timepoint post differentiation day 50. *Note*. Data are represented as Data ± SEM (box) unless mentioned. ***p < 0.0005. Detailed statistical information is provided in Supplementary Table 1. Source data

In addition to the added labeled α-Syn, cells also contain unlabeled, endogenous α-Syn. To test the effect of this on our FRET sensor, we aggregated 70 μM labeled (1:1 equimolar ratio of AF488 and AF594) α-Syn either in the absence or presence of 7 μM unlabeled WT α-Syn and characterized the aggregates using both single-molecule confocal and TIRF microscopy. Both the FRET and size distribution were unaffected by the presence of unlabeled α-Syn (Extended Data Fig. 4ai–4iii). We subsequently investigated whether different concentrations of endogenous (unlabeled) α-Syn would affect the FRET biosensor in human cells by applying AF488-labeled and AF594-labeled α-Syn to an isogenic series of iPSC lines with a range of SNCA alleles: SNCA null, SNCA 2 alleles and SNCA 4 alleles (Extended Data Fig. 4bi,bii). smFRET analysis of the size and FRET distribution of the labeled α-Syn aggregates in lysates, as well as the FRET in cell microscopy (Extended Data Fig. 4c,d), confirmed that there was a negligible effect of the endogenous α-Syn on the FRET signal.

Taken together, intracellular FRET, smFRET and TIRF microscopy can detect the initial stages of self-assembly, oligomer formation and structural conversion to proteinase-K-resistant Type-B oligomers inside cells. Aggregation inside cells is concentration and time dependent, and there is a reduced lag phase inside cells. Finally, there is an increased rate of oligomerization for A53T compared to WT and other mutants due, in part, to enhanced uptake and increased intracellular concentration of A53T.

### Oligomer formation occurs in ‘hotspots’ at varied cell locations

**Fig. 2:**
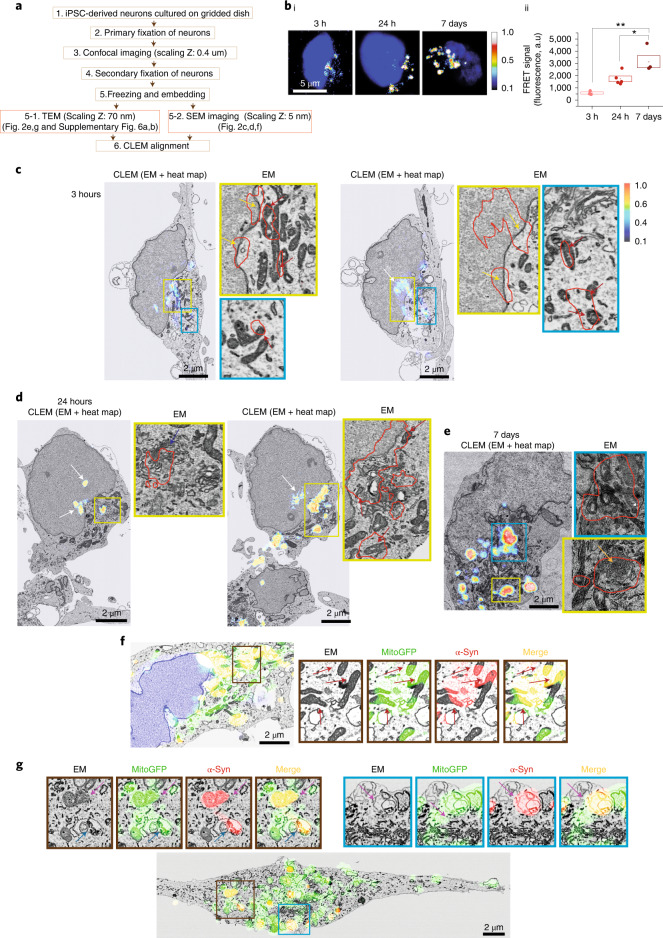
Oligomer formation occurs in multiple cell ‘hotspots’ at heterogeneous locations. **a**, Schematic workflow of the experimental steps for 3D-CLEM. The light imaging was performed at 0.4-μm intervals; the radial point spread function (PSF) (as given by $\lambda /2NA$, where *λ* is the excitation wavelength and NA is the numerical aperture of the objective lens) is 212 nm, and the axial PSF is approximately 500 nm. The EM section thickness is 5 nm, and the CLEM precision errors are shown in Extended Data Fig. 6. **bi**, FRET intensity heat maps showing aggregate formation with high FRET signal intensity at the core and low FRET intensity in the surrounding rim. **bii**, Intracellular FRET intensity increases after application of 500 nM A53T monomers (*n* = 3 independent experiments). **c**,**d**, Application of equimolar concentration of AF488-A53T α-Syn and AF-594-A53T α-Syn (total 500 nM); images were obtained at three different timepoints: 3 hours (**c**), 24 hours (**d**) and 7 days (**e**). Each panel is composed of a confocal image of CLEM (EM + FRET heat map) and zoom of EM alone. Colored arrows indicate aggregates at mitochondria (red), nucleus (white), membrane (yellow), Golgi apparatus (blue) and vesicles (orange). **f**,**g**, CLEM alignment using genetically engineered construct mitoGFP to label mitochondria (**f**: SEM and **g**: TEM). The red arrows indicate α-Syn detection within mitochondria. Note: Error maps for all images used for this figure are presented in Extended Data Fig. 5. Data are represented as data ± s.e.m. (box). *P < 0.05 and **P < 0.005. Detailed statistical information is shown in Supplementary Table 1. See also Extended Data Figs. 5 and 6. a.u., arbitrary units; PSF, point spread function. Source data

**Extended Data Fig. 5:**
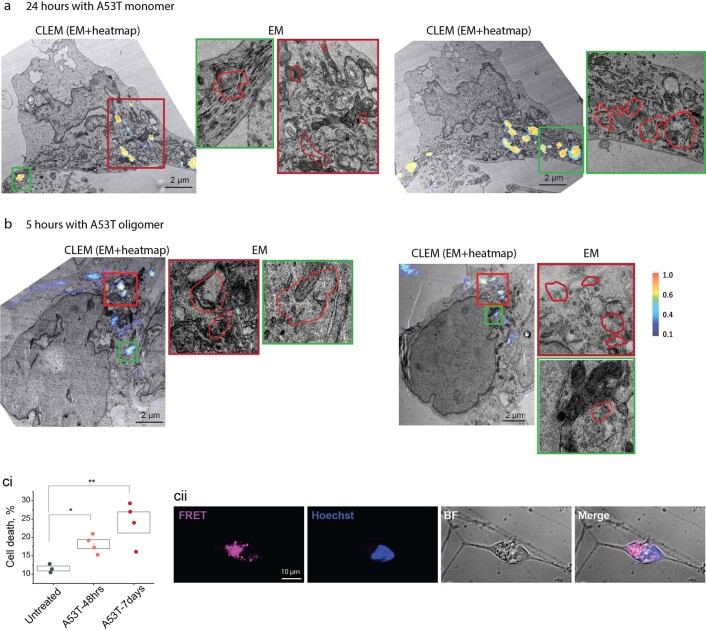
Related to main Fig. 2. **(a & b)** FRET-CLEM images after addition of AF488 and AF594-labeled α-Syn at 24 hours (A53T monomer) and 5 hours (A53T oligomer), respectively. High (red) and low (blue) areas of heatmap representing FRET intensity were detected in human neurons. **(c)** Cell death following treatment with A53T monomer in iPSC derived control neurons **(ci)** 18.2 ± 1.22% cell death after 48 hours and 24.1 ± 2.87% cell death after 7 days of treatment with A53T monomers in iPSC derived neurons. **(cii)** An example of cell selection for CLEM (after fixation). After 7 days of treatment, cells were fixed, and morphologically confirmed intact neurons were selected to perform CLEM experiment (n = 3 or 4 independent experiments). *Note*. Data are represented as Data ± SEM (box). *p < 0.05, **p < 0.005. Detailed statistical information is provided in Supplementary Table 1. Source data

To study the ultrastructural location of A53T oligomer formation in cells, we combined FRET imaging of labeled α-Syn with serial section electron microscopy (EM) and with focused ion beam milling combined with scanning electron microscopy (FIB-SEM) at 5-nm voxel resolution in human induced pluripotent stem cell (hiPSC)–derived neurons (Fig. 2a). We applied AF488-A53T α-Syn and AF594-A53T α-Syn monomer and oligomer to neurons, generated FRET signal heat intensity maps of aggregate formation (Fig. 2bi,bii and Extended Data Fig. 5a–c) and then tracked the same cells for EM.

**Extended Data Fig. 6:**
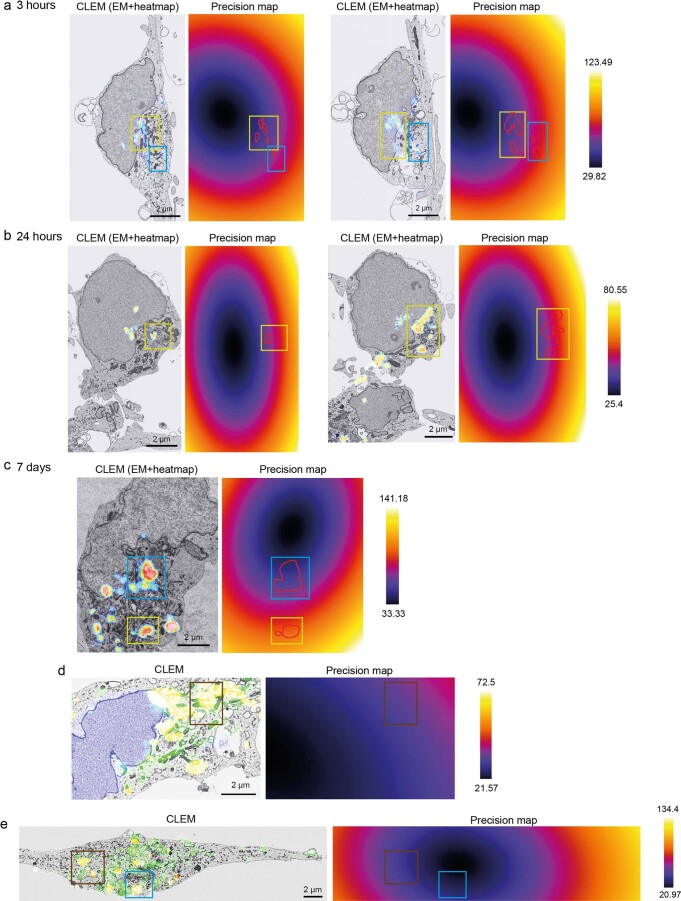
Related to main Fig. 2. **(a-e)** Global prediction of registration error in nanometers (nm) from Fig. 2c-g respectively. The CLEM precision error is 20 nm to 150 nm in the x.y axis, and importantly around 20 nm at the point of the alignment organelle (nucleus or mitochondria). Organelles such as autophagosomes, lysosomes and mitochondria are small in size (< 1.5 μm), whilst the nucleus is larger (<10μm). If the size of the organelle is larger relative to the precision error (at the point of alignment), the fluorescent signals are likely to originate within the identified subcellular region, as is the case for larger organelles, while the fluorescent signal may originate partially outside the EM imaging area for smaller organelles.

Integrated spatial information was obtained by aligning using a nuclear marker only. At 3-hour and 24-hour incubation, FRET-FIB-SEM shows that oligomer formation occurs in a range of localizations in the cell that encompass nucleus, mitochondria and cytosol (Fig. 2c,d and Extended Data Fig. 5a). After 7 days, the FRET signal, combined with serial section EM, showed that the maturing aggregates (7 days; Fig. 2e) expand to occupy regions of the cell that encompass several different organelles as well as the cytoplasm in between organelles. Error maps using nucleus alignment confirm an overall error of 20–141 nm (Extended Data Fig. 6a–c)^20^. To improve confidence in the localization, we adopted organellar alignment of light and EM images using a genetically modified construct to visualize mitochondria (mitoGFP) and integrated the mito-GFP and AF-594 α-Syn fluorescence with the EM images. We show co-localization of α-Syn and mitoGFP fluorescence in mitochondria and degradative pathways for mitochondrial components—for example, autophagosome-like structures—using both FIB-SEM (Fig. 2f) and transmission electron microscopy (TEM) (Fig. 2g). Error maps for mitochondria-aligned CLEM reveal the accuracy of 20–134 nm (Extended Data Fig. 6d,e).

Aggregation hotspots form in cellular spaces crowded with organelles and then mature in a stereotypical manner into an aggregate that contains highly ordered aggregates at the center and loosely ordered protein in the rim. Early aggregation of A53T occurs in multiple locations within the cell, which act as a ‘seed’ for aggregation, including nuclear membrane, mitochondria and vesicles. Multiple seeding events in human cells may contribute to the reduced lag phase of aggregation.

### Cardiolipin accelerates the oligomerization of A53T

**Fig. 3:**
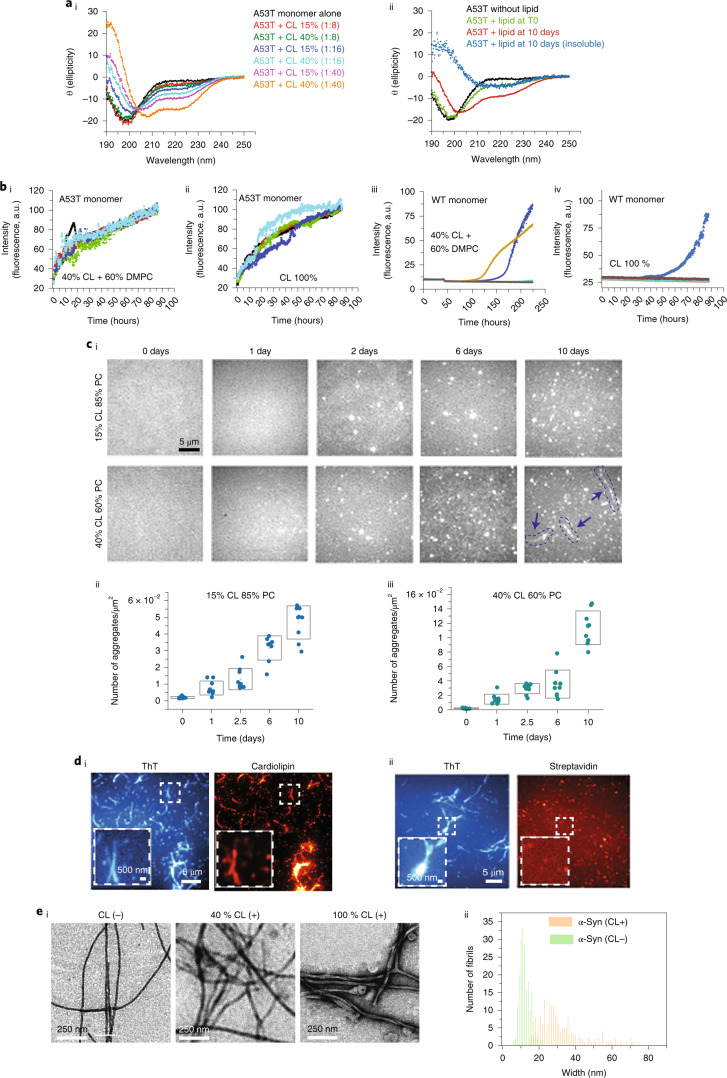
CL triggers and accelerates the aggregation of A53T α-Syn. **a**, Effect of CL on far-UV CD spectra of α-Syn. **ai**, A53T monomer (10 µM) in the presence of 15% and 40% CL at 1:8, 1:16 and 1:40 lipid:protein ratios. **aii**, A53T monomer in the absence of liposomes (black curve), in the presence of 40% CL before incubation (green curve), at plateau phase (red curve) and insoluble fraction (blue curve) after incubation at 37 °C in the presence of 40% CL and 60% PC liposomes with 1:8 ratio. The minima between 210 nm and 220 nm for the insoluble fraction shows the presence of amyloid structures in the sample. **bi**–**biv**, 50 µM of the A53T monomer led to a substantially fast increase in ThT fluorescence in the presence of 40% or 100% CL compared to WT monomer. **c**, Time-dependent SAVE images of A53T monomers incubated with 15% or 40% CL over 0–10 days show an increase in the number of aggregates over time. **ci**, Representative images. Red arrows indicate amyloid fibrils. **cii**,**ciii**, Quantification of the TIRF microscopy images. **di**,**dii**, TIRF microscopy analysis shows co-localization between CL and α-Syn fibrils (ThT positive). **ei**, TEM images show that, in the presence of CL, fibrils of α-Syn have different morphology. **eii**, Quantitative histogram of fibril width shows the large distribution of width in the presence of CL (100% CL), which is expected for a hierarchical self-assembly model of amyloid formation. A total of 200 fibrils were analyzed for each group using an Image-J plugin^54^. Note: Data are represented as mean ± s.d. Detailed statistical information is shown in Supplementary Table 1. See also Extended Data Fig. 7. a.u., arbitrary units; DMPC, dimyristoylphosphatidylcholine.

**Extended Data Fig. 7:**
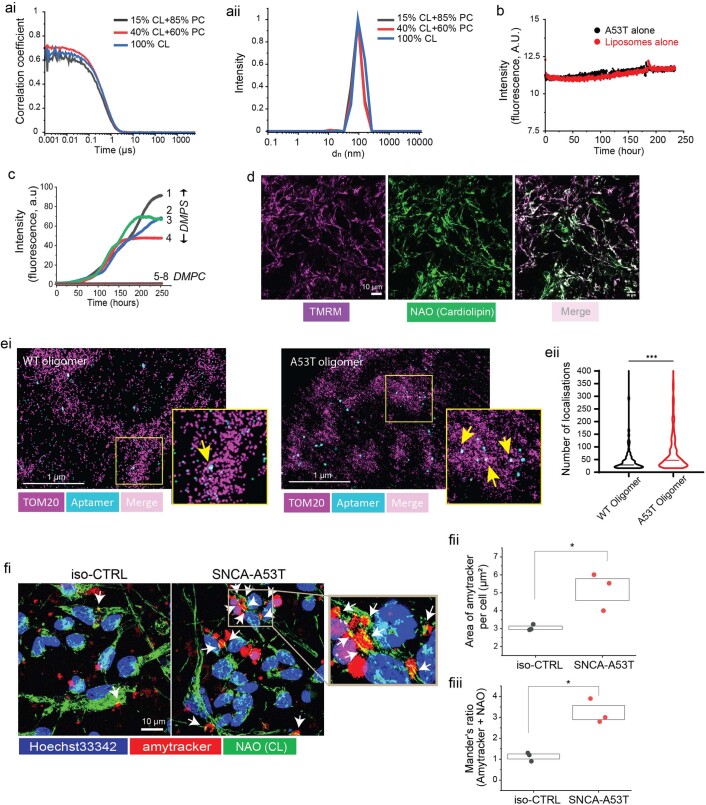
Related to main Fig. 3 & 4. **(a)** The hydrodynamic diameter (d_h_) of 150 mM lipid vesicles was determined by dynamic light scattering. **(ai)** Correlation function of the sample **(aii)** Size distribution of lipid vesicles determined by mass unweighted fitting which shows the distribution of lipid vesicles centered at 89, 92 and 104 nm for 15%, 40% and 100% cardiolipin in the vesicles, respectively. **(b)** No increase in ThT was observed in the control experiments with either A53T alone (black line) or liposomes alone (red line). **(c)** Kinetics of amyloid formation by α-Syn (50 µM) in the presence of DMPS (curve 1-4) and in the presence of DMPC (curve 5-8). Measurements were performed at 37 °C and pH 7.4. Protein concentration was 50 µM and lipid vesicle concentration was 400 µM. Thioflavin T (ThT) fluorescence was excited at 450 nm, and the emission wavelength was 482 nm. **(d)** NAO is co-localized mainly with a mitochondrial indicator, TMRM. **(ei & ii)** Single-molecule light microscopy (SMLM) images showing tom20-labeled mitochondria (dSTORM) and aggregates imaged with the aptamer (aptamer-DNA PAINT) in cyan, in WT and A53T oligomer treated iPSC-derived cortical neurons. Panel highlighted shows higher magnification. **(eii)** Quantification of the number of single-molecule localizations detected per aggregate in WT and A53T oligomer treated conditions (n = 70-310 aggregates per condition, *** *p* < 0.0005, Mann-Whitney t-test). **(f)** Contacts between α-Syn aggregates (labeled with amytracker) and cardiolipin (labelled with NAO) **(fi)** Representative images. **(fii)** Amytracker exhibits a higher fluorescent signal in SNCA-A53T than iso-CTRL neurons after the application of recombinant WT (99% monomer, 1% oligomer) (n = 3 fields imaged over 2 independent experiments). **(fiii)** Amytracker co-localization with NAO is higher in SNCA-A53T neurons than iso-CTRL (n = 3 fields imaged over 2 independent experiments). *Note*. Data are represented as Data ± SEM (box) unless mentioned. *p < 0.05, ***p < 0.0005. Detailed statistical information is provided in Supplementary Table 1. Source data

α-Syn was observed in the FRET-CLEM data in association with mitochondria. Cardiolipin (CL) constitutes 10–20% of the mitochondrial membrane^21,22^, and so we investigated the interaction between α-Syn and CL-containing 100-nm-sized lipid vesicles (Extended Data Fig. 7ai,aii). Circular dichroism (CD) spectroscopy showed that α-Syn first adopts an α-helical conformation in the presence of CL-containing vesicles, as previously reported (Fig. 3ai)^23^. This change depends on the CL content in the vesicles and the lipid:protein molar ratio. With higher CL content and a higher lipid:protein molar ratio, we detected an increase in the α-helical structure (Fig. 3ai). Over time, CD spectroscopy (40% CL liposomes; 8:1 lipid:protein ratio) revealed a transition from α-helix to a mixture of protein structures (Fig. 3aii). Centrifugation and CD analysis of the pellet showed significant β-sheet content, as evidenced by a minimum at 210–220 nm (ref. ^24^). These data confirm that α-Syn interacts with CL and forms secondary structures (α-helical) initially but, over time, can acquire β-sheet-rich content.

Next, we followed the formation of amyloid structure using the amyloid-binding dye thioflavin-T (ThT). A53T monomer exhibited rapid aggregation, with an absence of the lag phase observed as a sharp increase in the ThT fluorescence (Fig. 3bi,bii) in the presence of CL liposomes (40–100% CL, with phosphatidylcholine (PC), which alone does not induce aggregation (Extended Data Fig. 7b,c; 8:1 lipid:protein ratio)). A long and variable lag phase was observed in 40–100% CL and WT α-Syn (Fig. 3biii,biv). Single aggregate visualization by enhancement (SAVE) imaging with ThT (Fig. 3ci) showed that, in 15% or 40% CL (8:1 lipid:protein), aggregates formed over 10 days of incubation (Fig. 3cii,ciii). Forty percent CL led to the formation of large fibrillar aggregates after 10 days. Using biotinylated CL (100% CL, 8:1 lipid:protein ratio), we simultaneously visualized CL using Alexa Fluor 647 (AF647)–tagged streptavidin and aggregates using ThT, showing that amyloid fibrils contained CL, the percentage coincidence between ThT and CL using A53T monomer was 84% (95% confidence interval (CI), 73%, 106%), and using control (non-biotinylated lipid) it was 0.6% (95% CI, 0.3%, 3.5%) (Fig. 3di,dii). Our SAVE imaging data show that the interaction of A53T α-Syn and lipid vesicles results not only in extraction of CL but also in the incorporation of CL into the amyloid fibrils. We then studied the morphology of the fibrils formed in the presence of CL (8:1 lipid:protein ratio) by TEM and found that fibrils generated are markedly different from those formed in the absence of CL, showing helical periodicity along the length of fibrils and a greater number of protofilaments (Fig. 3ei,eii). Thus, CL vesicles may promote the lateral association of α-Syn protofilaments, which is consistent with the hierarchical self-assembly model of amyloid fibrils^25^.

**Fig. 4:**
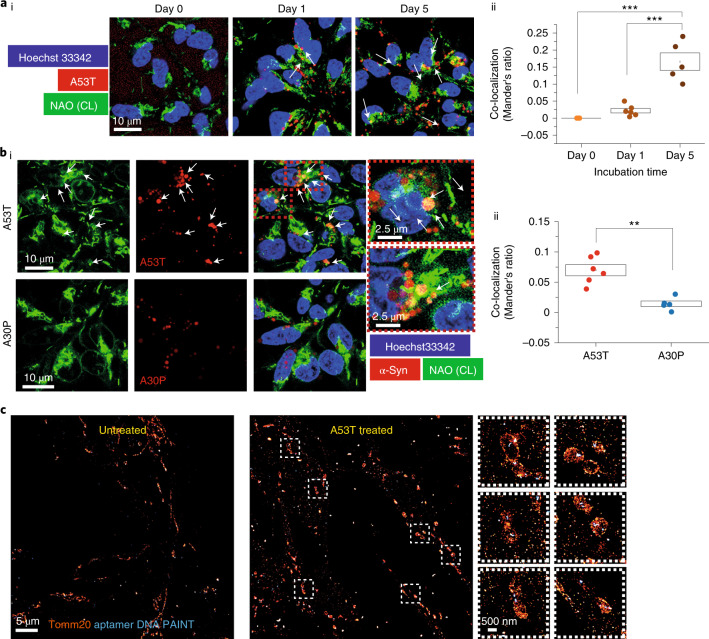
A53T α-Syn contacts CL as it aggregates. **ai**,**aii**, Visualization of α-Syn contacts with CL in hiPSC-derived neurons using NAO. hiPSC-derived neurons were treated with 1 μM AF488-A53T α-Syn monomer, and contacts were measured at three timepoints: time 0, day 1 and day 5 (*n* = 5 fields imaged). **bi**,**bii**, Total α-Syn co-localisation with CL was higher in the cells treated with A53T monomers than with A30P monomers (*n* = 4 or 6 fields imaged). **c**, SMLM images show mitochondria labeled with Tomm20 (dSTORM) and aggregates labeled with a DNA-based aptamer (aptamer DNA PAINT). Panels on the right show a higher magnification. Note: Data are represented as data ± s.e.m. (box). **P < 0.005 and ***P < 0.0005. Detailed statistical information is shown in Supplementary Table 1. See also Extended Data Fig. 7. Source data

Inside cells, we visualized endogenous CL using a fluorescent probe, nonyl acridine orange (NAO; Biotium, 70012), which co-localized with another mitochondrial indicator, tetramethylrhodamine methyl ester (TMRM) (Extended Data Fig. 7d). Control iPSC-derived neurons were treated with 1 uM AF488-A53T α-Syn or AF488-A30P α-Syn monomers for 48 hours. AF594 A53T α-Syn co-localizes with endogenous CL increasingly over time (Fig. 4a) and to a lesser degree than AF594 A30P a-Syn (Fig. 4b). We used super-resolution microscopy (aptamer DNA-PAINT to detect α-Syn aggregates and direct stochastic optical reconstruction microscopy (dSTORM)^26^) to show the presence of aggregates at the mitochondria (Extended Data Fig. 7e). To capture aggregate formation by both the externally applied α-Syn and the endogenous α-Syn, we used the dye amytracker, which binds specifically to β-sheet structures of amyloid aggregates. iPSC-derived neurons treated with WT oligomer exhibited increased aggregate formation (labeled with amytracker) in SNCA-A53T cells compared to isogenic control (iso-CTRL) and increased co-localization of those aggregates with CL (Extended Data Fig. 7fi–fiii).

Taken together, these data suggest that CL can rapidly trigger the aggregation of A53T, and CL is then incorporated into the aggregating structure of α-Syn, which may further accelerate the aggregation process.

### A53T impairs mitochondrial bioenergetics and induces mitochondrial dysfunction

**Fig. 5:**
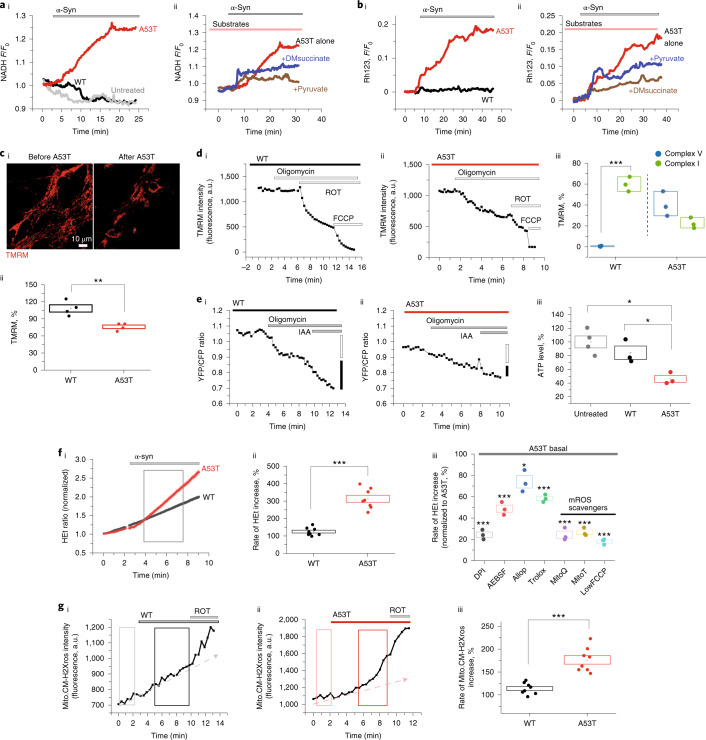
A53T α-Syn impairs mitochondrial bioenergetics and induces mitochondrial dysfunction. **ai**, Increase in NADH autofluorescence after application of 500 nM A53T α-Syn (normalized to 1). **aii**, The increase in NADH is prevented by pre-incubation with pyruvate and succinate. **bi**, A53T monomer depolarizes Δψ_m_ as measured by an increase in rhodamine 123 (Rh123) fluorescence. **bii**, The decreased Δψ_m_ is also reversed by pre-application of pyruvate and succinate. **ci**,**cii**, Images showing reduction in Δψ_m_ after 30-minute incubation with A53T compared to WT and the quantitative histogram (*n* = 4 independent experiments). **di**–**diii**, Response of Δψ_m_ to complex V inhibitor (oligomycin: 2.4 μg ml^−1^), complex I inhibitor (rotenone (ROT): 5 μM) and mitochondrial uncoupler (FCCP: 1 μM). The basal fluorescence intensity was reset at 1,500–2,500 a.u. (*n* = 3 independent experiments). **ei**–**eiii**, A53T reduces the total ATP production measured by FRET-ATP sensor compared to WT-treated or untreated cells (*n* = 3 or 4 independent experiments). **fi**,**fii**, Superoxide was increased after application of A53T but not WT α-Syn (*n* = 8 independent experiments). **fiii**, Inhibition of A53T-induced ROS by different inhibitors (*n* = 3 independent experiments). **gi**–**giii**, mROS production was increased by A53T (*n* = 8 independent experiments)*.* Note: 500 nM α-Syn monomer was applied for each experiment unless otherwise mentioned. Note: Data are represented as data ± s.e.m. (box). *P < 0.05, **P < 0.005 and ***P < 0.0005. Detailed statistical information is shown in Supplementary Table 1. See also Extended Data Fig. 8. a.u., arbitrary units. Source data

**Extended Data Fig. 8:**
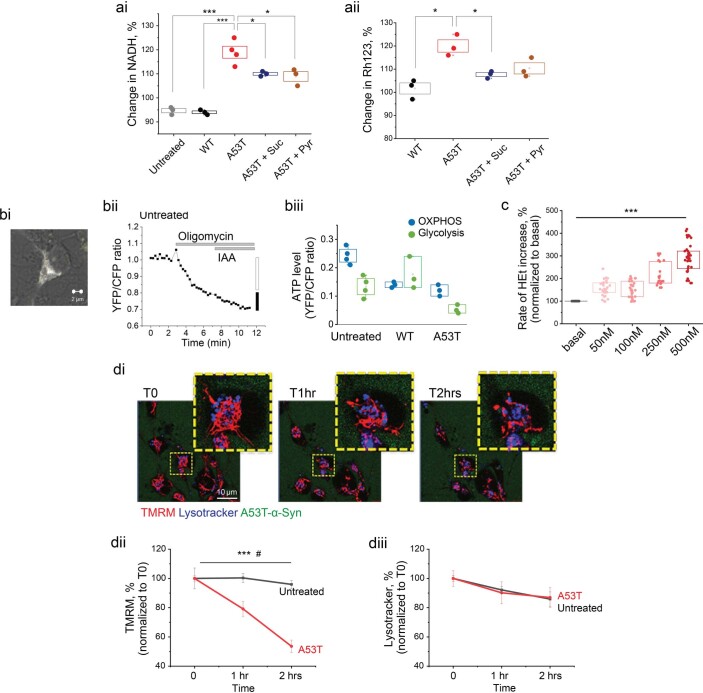
Related to main Fig. 5. **(ai & ii)** Quantitative histograms referring to Fig. 4a and b respectively Suc; DMsuccinate, Pyr; Pyruvate, Rh123; Rhodamine 123. **(ai)** A53T vs untreated or WT or A53T + Suc or A53T + Pyr (n = 3 or 4 fields imaged over 2 independent experiments). **(aii)** A53T vs WT or A53T + Suc (n = 3 independent experiments) **(bi)** Representative image of transfected cells with ATP probe. **(bii)** Representative trace of ATP measurement in untreated cells. **(biii)** The proportional contribution of oxidative phosphorylation (OXPHOS) and glycolysis to ATP levels in untreated, WT or A53T treated cells, shown in main Fig. 5eiii. **(c)** Superoxide is produced in a concentration dependent manner from A53T-α-Syn monomer (n = 32, 34 or 37 cells over 2 independent experiments). **(di)** Time lapse single cell imaging of mitochondria after application of AF488-A53T α-Syn. **(dii & iii)** Δψ_m_ measured by TMRM fluorescent signal (intensity, a.u.) is significantly reduced after 2 hours whilst there is no change in the fluorescent signal (intensity, a.u.) of lysotracker after application of A53T α-Syn (n = 3 cells). *Note*. Data are represented as Data ± SEM (box) unless mentioned. *p < 0.05, ***p < 0.0005. Detailed statistical information is provided in Supplementary Table 1. Source data

We investigated the functional consequence of A53T and WT α-Syn monomers applied exogenously to primary rodent neurons. Autofluorescence of complex I substrate NADH was used to measure cellular redox state and complex I function. We found that 500 nM A53T induced an increase in NADH fluorescence (119 ± 2.48%; Fig. 5ai and Extended Data Fig. 8ai), suggesting an inhibition of complex I function similar to the effect of oligomeric α-Syn^27^. A53T-induced complex I inhibition was associated with mitochondrial depolarization (120.4 ± 2.6%; Fig. 5bi and Extended Data Fig. 8aii), measured using rhodamine 123 fluorescence. NADH fluorescence could be partially restored by pre-incubation of cells with substrates for complex I (5 mM pyruvate) or complex II (membrane-permeable analog of succinate-dimethyl succinate (5 mM DMsuccinate)), showing that respiratory chain function could be rescued (108.9 ± 3.48% by pyruvate, 110 ± 0.5%; Fig. 5aii and Extended Data Fig. 8ai) and that improvements in the respiratory chain function also restore the mitochondrial membrane potential (Δψ_m_) (107 ± 0.88% by DMsuccinate; Fig. 5bii and Extended Data Fig. 8aii). A53T reduced Δψ_m_, measured by TMRM, after 30 mininutes (76.1 ± 3.1%), whereas Δψ_m_ was unchanged in WT monomer-treated cells (108.25 ± 6.30%; Fig. 5c). We investigated the decreased Δψ_m_ by testing its sensitivity to complex I and V inhibition. In healthy mitochondria in WT-treated cells, Δψ_m_ is maintained predominantly through the action of complex I–dependent respiration (reduction by 59.8 ± 4.04% in Δψ_m_ after rotenone but only 0.43 ± 0.25% reduction by the complex V inhibitor oligomycin). However, A53T-treated cells exhibited only 22.4 ± 2.94% reduction of Δψ_m_ after complex I inhibition but 40.3 ± 6.85% reduction by complex V (Fig. 5di–diii). Therefore, in cells exposed to the A53T mutant, the Δψ_m_ cannot be maintained sufficiently through respiration and must use complex V (in reverse mode as an ATPase) to maintain it. A53T-treated cells exhibited significantly reduced ATP production than WT-treated cells (Fig. 5eiii and Extended Data Fig. 8b).

We found that 500 nM A53T increased the rate of superoxide production in contrast to WT (Fig. 5fi,fii). The generation of cytosolic ROS was dependent on the concentration of applied monomer (Extended Data Fig. 8c). We investigated the source of the A53T-induced ROS using a range of inhibitors: mito-TEMPO or mitoQ (mitochondria-targeted antioxidants); Trolox (a water-soluble analog of vitamin E); and diphenyleneiodonium chloride (DPI) or 4-(2-aminoethyl)-benzolsulfonylfluorid-hydrochloride (AEBSF) (inhibitors of NADPH oxidase (NOX)). Mitochondrial ROS (mROS) scavengers effectively blocked A53T-induced ROS, suggesting that mROS may be a major source of excess ROS (Fig. 5fiii), with additional activation of NADPH oxidase. A53T monomer induced higher levels of mROS (176.6 ± 9.3% of basal) compared to WT (113.8 ± 4.4% of basal) (Fig. 5gi–giii). Time-lapse imaging of mitochondria within cells reveals the kinetics of uptake and mitochondrial dysfunction induced by A53T monomer application (Extended Data Fig. 8di,dii).

### A53T α-Syn induces mitochondrial permeability transition pore opening

**Fig. 6:**
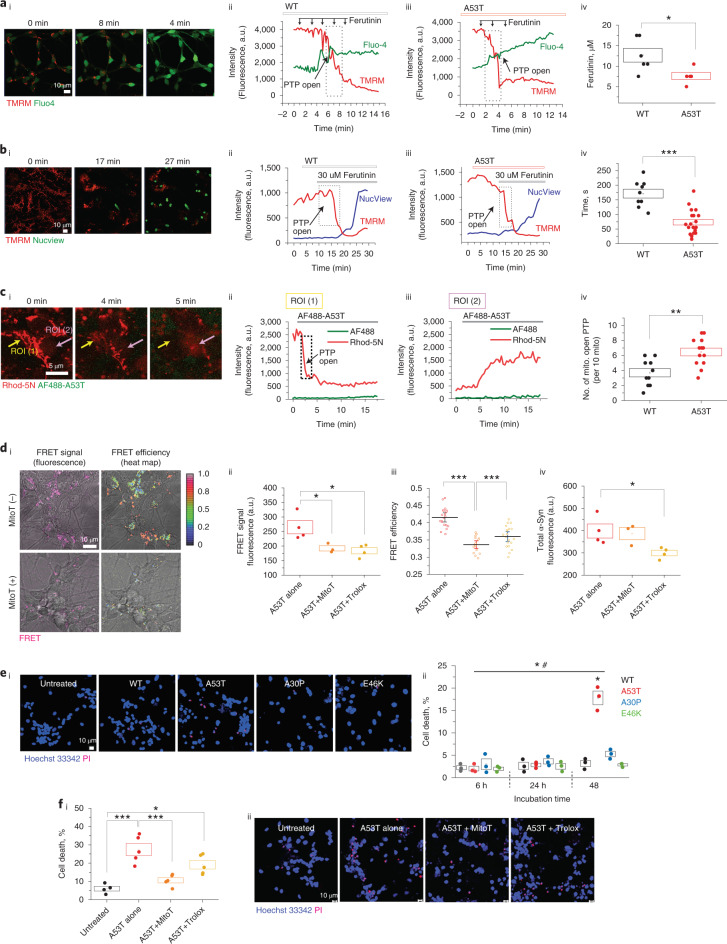
A53T α-Syn induces mPTP opening, and mROS accelerates oligomerization and cell death. **ai**, Representative time course images showing that Δψ_m_ reduction (TMRM) is followed by an increase of cytoplasmic calcium level (Fluo-4) at the point of mPTP opening. **aii**,**aiii**, Representative traces from the cells treated with 500 nM of WT or A53T α-Syn, respectively. **aiv**, A53T-treated cells require lower concentrations of ferutinin to open the mPTP than WT-treated cells (*n* = 4 or 6 independent experiments). **bi**, Representative time course images showing that apoptosis (NucView) is induced after a substantial loss of Δψ_m_ after ferutinin-induced PTP opening. **bii**,**biii**, Representative traces and WT-treated or A53T-treated cells. **biv**, A53T α-Syn treatment induces earlier PTP opening than WT α-Syn (*n* = 9 or 19 cells over two independent experiments). **c**, mPTP opening in isolated mitochondria from permeabilized cells. **ci**, Representative time course images of mPTP opening after applying AF-488-A53T α-Syn. **cii**,**ciii**, The mitochondrial area (ROI 1 area) exhibited a rapid loss of Δψ_m_, whereas the extra-mitochondrial area (ROI 2) exhibited increased intensity of Rhod-5N after mPTP opening. **civ**, Quantitative histogram showing that PTP opening occurs earlier in A53T-treated than WT-treated mitochondria (*n* = 10 or 13 cells over two independent experiments). **d**, FRET intensity and FRET efficiency of A53T are reduced by treatment with mito-TEMPO. **di**, The representative images. **dii**,**diii**, Mito-TEMPO-treated cells show reduced A53T FRET intensity (**dii**; *n* = 3 or 4 independent experiments) and efficiency (**diii**; *n* = 20 or 15 cells over three independent experiments, and error bars represent 95% CIs). **div**, Application of Trolox to cells reduced FRET intensity signal by reducing uptake of donor (**dii**–**div**; *n* = 3 or 4 independent experiments). **ei**,**eii**, Cell death was induced by 48-hour incubation of A53T but not by WT or A30P/E46K (*n* = 3 independent experiments). **fi**,**fii**, A53T-induced cell death was rescued by treatment with mito-TEMPO (*n* = 4 or 5 independent experiments). Note: 100 μM Trolox and 0.5 μM mito-TEMPO (MitoT) were pre-treated 30 minutes before α-Syn application. Note: Data are represented as data ± s.e.m. (box). *P < 0.05, **P < 0.005 and ***P < 0.0005. Detailed statistical information is shown in Supplementary Table 1. a.u., arbitrary units. Source data

We previously showed that early opening of mitochondrial permeability transition pore (mPTP) mediates α-Syn oligomer-induced cell toxicity^12^. To test whether A53T α-Syn affects mPTP opening, cells were loaded with TMRM and the cytosolic calcium dye Fluo-4, followed by stepwise application of ferutinin, an inducer of mPTP opening by mitochondrial calcium overload^28^ (reviewed in ref. ^29^). A53T α-Syn lowered the threshold of mPTP opening (Fig. 6aiv). The latency to mPTP opening (rapid loss of TMRM fluorescence) after a high concentration of ferutinin was also reduced (Fig. 6bii–biv), and we observed that caspase 3–dependent apoptosis was induced in all cells that exhibited mPTP opening.

We measured mPTP opening in isolated mitochondria of permeabilized cells. Cells were loaded with Rhod-5N and permeabilized with 40 µM digitonin in pseudo-intracellular solution^30^. Application of A53T monomers induced a rapid loss of Rhod-5N as the PTP opened and the dye left the mitochondria (ROI 1; Fig. 6ci,cii), resulting in increased Rhod-5N fluorescence in the cytoplasm (extra-mitochondrial region, ROI 2; Fig. 6ci,ciii). The number of mitochondria that exhibited mPTP opening within 15 minutes was higher in the A53T-treated mitochondria compared to WT (Fig. 6civ), in line with the results from the whole cell model.

mPTP opening requires the structural conformation of α-Syn to a β-sheet-rich oligomer^12^. Therefore, A53T monomers, in contact with mitochondrial membrane, rapidly form oligomeric species that are able to open the mPTP and induce apoptosis and cell toxicity.

### A53T-induced mROS accelerates oligomerization and cell death

A53T α-Syn generates mROS, and we investigated whether mROS alters aggregation. Oligomer formation (FRET signal) was measured after co-treating with Trolox or mito-TEMPO, a mitochondria-targeted antioxidant. As seen in Fig. 6di–div, the application of 100 μM Trolox reduced uptake of the A53T monomer into cells (Fig. 6div), reducing intracellular FRET intensity (Fig. 6dii), but with no effect on structural conversion (FRET efficiency)(Fig. 6diii). In contrast, the mitochondrial-targeted antioxidant mito-TEMPO resulted in a significant reduction in both FRET intensity (Fig. 6dii) and efficiency (Fig. 6diii) with preservation of the donor intensity (that is, preservation of uptake into cells but reduced oligomerization). Thus, generation of mROS is a key factor in the intracellular oligomerization of the A53T α-Syn, and scavenging mROS modulates the formation of aggregates. A53T monomer treatment caused increased cell death (Fig. 6ei,eii) after 48 hours, and A53T-induced cell death was effectively inhibited by mito-TEMPO (Fig. 6fi,fii).

These results suggest that impaired respiration and generation of mROS act synergistically with CL to drive the intracellular oligomerization of the A53T monomer and contribute to neuronal death.

### Cellular phenotypes in SNCA-A53T hiPSC-derived neurons

**Fig. 7:**
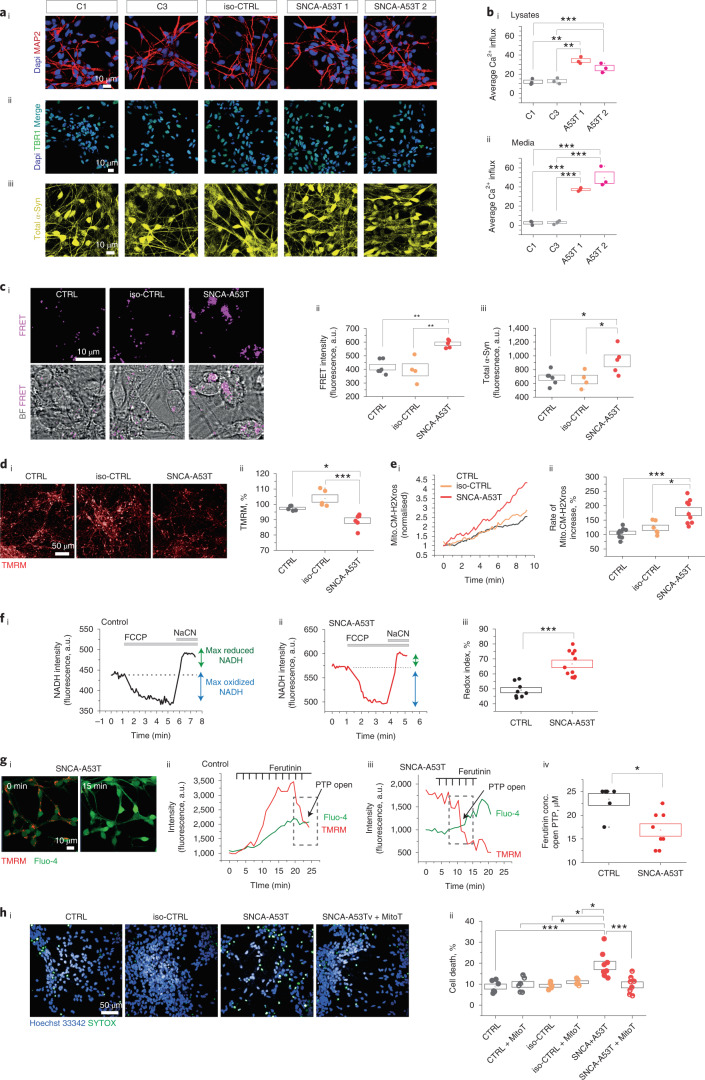
SNCA-A53T hiPSC-derived neurons exhibit accelerated α-Syn seeding and mitochondrial dysfunction. **a**, Characterization of cortical neurons using immunocytochemistry at day 70 of neural induction (C1: control; C2: control, iso-CTRL: isogenic control of SNCA-A53T mutant line 1; A53T 1: SNCA-A53T mutant line 1; A53T 2: SNCA_A53T mutant line 2). **ai**, Neuronal marker MAP2. **aii**, Cortical layer VI marker TBR1. **aiii**, Total α-Syn. Quantification is shown in Extended Data Fig. 10ai–aiii. **bi**,**bii**, Lysates from SNCA-A53T neurons contain oligomers that cause increased membrane permeability compared to control neurons (*n* = 3 independent experiments). **ci**–**ciii**, Application of AF-488 A53T and AF594-A53T α-Syn to cells results in higher intracellular FRET intensity (*n* = 4, 5 or 6 independent experiments). **di**,**dii**, There is lower Δψm in A53T-SNCA neurons than in control, measured by TMRM fluorescence (*n* = 5 or 6 independent experiments). **ei**,**eii**, Increased production of mROS measured by mitoTrackerCM-H2Xros in SNCA-A53T neurons (*n* = 7 or 9 independent experiments). **fi**–**fiii**, SNCA-A53T neurons exhibit a higher redox index than control neurons, indicating complex I inhibition (*n* = 8 or 11 independent experiments). Data from individual lines are present in Extended Data Fig. 10b. **gi**–**giv**, SNCA-A53T require lower concentrations of ferutinin for mPTP opening. Data from individual lines are present in Extended Data Fig. 10c (*n* = 6 or 8 independent experiments). **hi**,**hii**, SNCA-A53T neurons exhibit higher cell death than control neurons at day 80 (there was no difference in basal cell death at day 60 as shown in Extended Data Fig. 10d), which can be rescued by 0.1 mM mito-TEMPO (*n* = 5 or 9 independent experiments). Note: Data are represented as data ± s.e.m. (box). *P < 0.05, **P < 0.005 and ***P < 0.0005. Detailed statistical information is shown in Supplementary Table 1. See also Extended Data Fig. 10. a.u., arbitrary units; BF, bright-field. Source data

**Extended Data Fig. 9:**
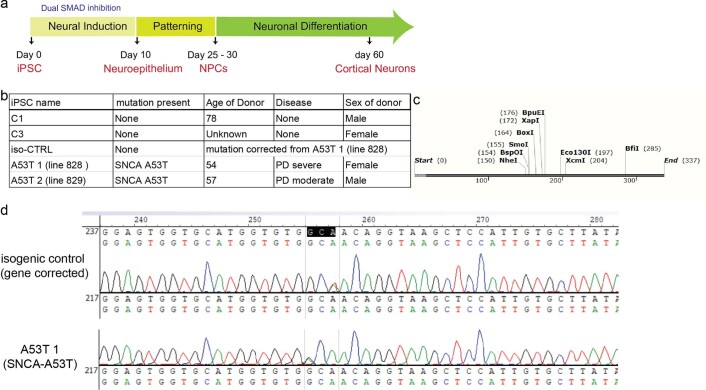
Related to main Fig. 7. **(a)** A summary of the neuronal differentiation protocol from hiPSCs into cortical neurons. Enrichment of hiPSC- derived cortical neurons is achieved after day 60. **(b)** Donor information of iPSCs used in this study. **(c & d)** Design for Sanger sequence to confirm A53T point mutation on SNCA and isogenic control line. Sanger sequencing confirmed that clone #2-68 and #5-26 are homozygous for Threonine at both alleles (GCA or GCT), while their isogenic clones and c1729 lines (iPSC derived from PD patient carrying A53T mutation) are heterozygous for Alanine (GCA) and T (ACA) at each allele.

**Extended Data Fig. 10:**
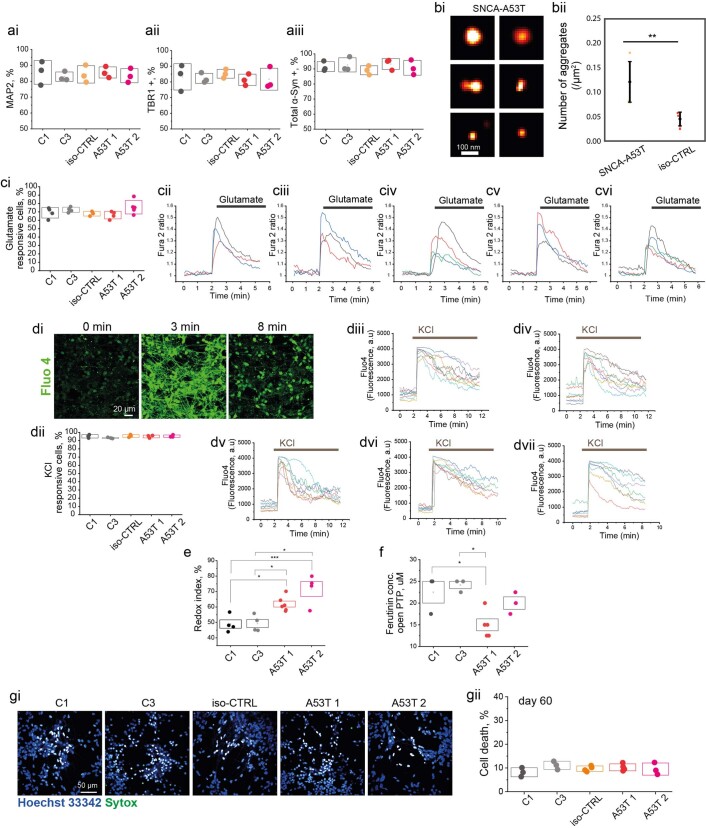
Related to main Fig. 7. **(ai-iii)** Quantification of neuronal markers and cells expressing total α-Syn for the main Fig. 7ai-iii (n = 3 independent experiments). **(b)** Aptamer PAINT on cell lysates from iso-CTRL and A53T show higher numbers of endogenous aggregates in A53T cells. **(bi)** Example AD-PAINT images of aggregates from SNCA-A53T. **(bii)** There is a two-fold increase in aggregates in cell lysates from SNCA-A53T compared to iso-CTRL (n = 2 independent experiments). Data expressed as mean ± SD. **(ci)** Assessment of glutamatergic signaling using [Ca^2+^] imaging. Both control and *SNCA-*A53T cells were responsive to 5 uM glutamate (n = 3, 4 or 5 independent experiments). **(cii-vi)** Representative traces of C1, C3, iso-CTRL, A53T 1 and A53T 2 lines respectively. **(d)** Assessment of calcium response to KCl using Fluo4 calcium indicator. **(di)** Representative images of Fluo4 changes (C1 line) in response to KCl. **(dii)** Both controls and SNCA-A53T lines have a high proportion of cells responding to KCl (n = 3 independent experiments). **(dii-vii)** Representative traces of C1, C3, iso-CTRL, A53T 1 and A53T 2 lines respectively. **(e)** Quantification of redox index for individual cell lines (n = 3 or 6 independent experiments) **(f)** Quantification of ferutinin concentrations to open PTP for individual lines (n = 3, 4 or 5 independent experiments). **(gi-ii)** Assessment of basal cell death at day 60 in vitro for individual cell lines. There is no significant difference across the lines (n = 3, 4 or 5 independent experiments). *Note*. Data are represented as Data ± SEM (box) unless mentioned. *p < 0.05, ***p < 0.0005. Detailed statistical information is provided in Supplementary Table 1. Source data

SNCA-A53T mutation results in early-onset disease with synucleinopathy in cortical and midbrain structures. We generated cortical neurons from hiPSCs from two patients carrying the A53T mutation (SNCA-A53T), an iso-CTRL and two healthy volunteers (CTRL), characterized in Extended Data Figs. 9 and 10. To measure endogenous oligomer formation in these lines, cell lysates were applied to an ultra-sensitive assay (single vesicle-based membrane permeabilization assay) that measures the ability of an aggregate to permeabilize membranes and induce calcium influx. The SNCA-A53T iPSC-derived neurons accumulate (Fig. 7bi) and secrete higher numbers of endogenous oligomeric species (Fig. 7bii), with permeabilizing capability, compared to control neurons. Endogenous aggregates are higher in cell lysates from SNCA-A53T compared to iso-CTRL using aptamer DNA PAINT^13^(Extended Data Fig. 10bi,bii).

We evaluated seeding and oligomerization in the SNCA-A53T neurons by measuring the FRET signal after adding AF488-A53T α-Syn and AF594-A53T α-Syn monomer to neurons. SNCA-A53T neurons showed both higher intracellular FRET signal (Fig. 7ci,cii) and total α-Syn uptake (Fig. 7ciii) compared to control cells, showing that the endogenous background of A53T α-Syn results in more rapid uptake and oligomerization of externally applied A53T α-Syn. SNCA-A53T neurons exhibit abnormal mitochondrial function, similarly to that observed upon a direct application of the A53T α-Syn: a reduction in Δψ_m_ (Fig. 7di,dii), higher production of mROS (Fig. 7ei,eii), complex I inhibition (Fig. 7fi–fiii and Extended Data Fig. 10e) and early PTP opening (Fig. 7gi–giv and Extended Data Fig. 10f). Lastly, the A53T neurons exhibited increased basal cell death that was prevented with 0.1 µM mito-TEMPO (Fig. 7hi,hii).

Cortical neurons derived from SNCA A53T patients with PD display higher levels of oligomer formation, associated with bioenergetic defects and oxidative stress. This results in accelerated seeding, oligomerization, PTP opening and cell death.

## Discussion

**Fig. 8:**
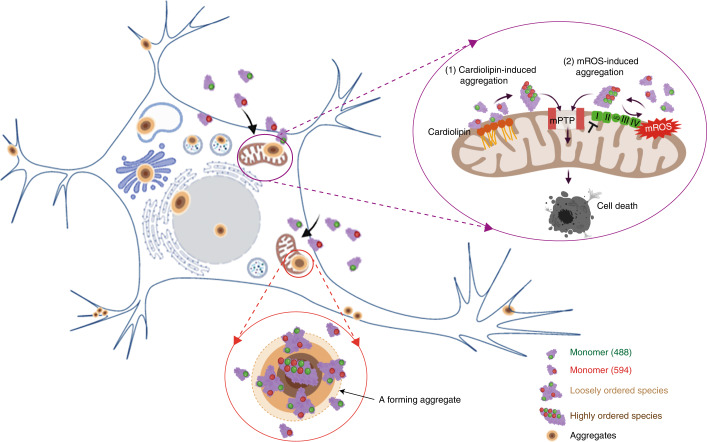
Graphical illustration showing how α-Syn monomers form aggregates inside neurons and induce cell toxicity. Graphical illustration showing how α-Syn monomers form aggregates inside neurons and induce cell toxicity. Monomeric α-Syn is taken up in neurons where it begins to self-assemble first into a population of amorphous, loosely ordered oligomeric species, which progress to form highly ordered oligomeric species. Aggregates form with a dense central core of highly ordered oligomers surrounded by a rim of loosely packed oligomers and occur in multiple hotspots throughout the cell body, including the nucleus, Golgi, vesicles and mitochondria. Mitochondria are a critical site of aggregation due to the functional consequences: CL triggers oligomerization of A53T α-Syn. A53T α-Syn induces over-production of mROS, promoting oligomerization of α-Syn. A53T α-Syn oligomerization impairs complex I function and ATP production and promotes early opening of mPTP, leading to cell death.

Precise characterization of the dynamic process of protein aggregation within human cells is a major challenge, leaving the mechanisms that trigger the initial stages of self-assembly and early aggregation in disease largely unknown. Our study integrated high-resolution biophysical approaches with hiPSC biology to comprehensively characterize the spatial and temporal features of protein aggregation in the intracellular environment. The A53T mutation drives the formation of the oligomeric structure, and, because the kinetics are accelerated compared to WT, this provided a tractable system to study the mechanism of structural conversion from monomers to oligomers and their toxic consequences (Fig. 8). Here we show that seeding on the membranes is the major driver in human neurons. Webelieve that the importance of this work lies in trying to understand how seeding occurs at the start of the disease process (before fibril formation).

The kinetics of α-Syn aggregation has emerged largely from in vitro experiments^10,16,17,31^. Aggregation commences with a lag phase due to the difficulty of monomers assembling to form oligomers alone (primary nucleation), followed by a rapid growth phase in which conversion is accelerated due to the relative ease of adding monomers to existing seeds, or oligomers, to form protofilaments and fibrils (elongation). Single-molecule fluorescence methods, including single-molecule confocal measurements of FRET efficiencies, have been used to precisely determine the structure of protein assemblies in in vitro systems. Type-A oligomers have low FRET efficiency, whereas β-sheet-structured, compact, Type-B oligomers have close stacking of fluorophores and high FRET efficiencies. In its original implementation, Cremades et al.^10^ showed that the Type-B oligomers (displaying a high FRET efficiency) were more resistant to proteinase K digestion and were also more cytotoxic than the Type-A (low FRET efficiency) oligomers. In a follow-up paper, Chen et al.^32^ were able to purify subtypes of oligomers, which they characterized using an array of biophysical techniques, including atomic force microscopy (AFM), Fourier-transform infrared spectroscopy (FTIR), cryo-EM and CD spectroscopy, and also showed that the FRET efficiency readout was able to discriminate between the subtypes of oligomers.

Traditionally, kinetic models of aggregation do not consider the complexity of the cellular environment, with the presence of cellular organelles, lipid surfaces, altered pH and protein degradation systems^15,33^. In this study, we applied both in-cell FRET and smFRET to directly detect and characterize the kinetics of formation, interconversion and accumulation of oligomers from the monomeric state in the native human cellular environment. smFRET was able to provide estimations of the size (deduced from fluorescence intensities) and structural group (deduced from FRET efficiencies) for each individual oligomeric species detected^10,34^. Inside human neurons, the monomeric population begins to rapidly self-assemble. We observed two distinct populations - amorphous oligomers, termed Type-A oligomers (characterized by low FRET efficiency), which were loosely packed and the first to form. These progressed to form Type-B oligomers (characterized by higher FRET efficiency), which were more compact and had a higher-order β-sheet structure over days. The kinetics of self-assembly and oligomer formation were dependent on the initial concentration of monomer, the time inside the cells and the sequence of the protein, in keeping with the known critical factors that affect aggregation in vitro.

The A53T mutation alters the kinetics of intracellular aggregation, with abolition of the lag phase, resulting in immediate self-assembly into small oligomers and rapid accumulation of more Type-B oligomers, in line with data^2,17^. The kinetic profile of aggregation in cells is likely due to the abundant protein–lipid interactions inside the cell. α-Syn is disordered in solution but can adopt an α-helical conformation upon binding to lipid membranes. The interaction of α-Syn with lipids at membrane surfaces can trigger the conversion of α-Syn from its soluble state into an aggregated state, enhance the rate of nucleation and significantly reduce the lag phase^35^. Lipid-induced generation of fibrils is highly sensitive to the specific sequence of the protein, particularly the region encompassing the residues 46–51, and the rate of lipid-induced aggregation is significantly higher in A53T compared to WT protein^36^. Our data suggest that human neurons, rich in multiple membrane surfaces and organelle interfaces, trigger the aggregation of A53T by stimulating primary nucleation and that the lipid–protein interaction is heavily dependent on the sequence of the protein, with A53T enhancing the lipid interaction.

CLEM is an integrated approach that closes the spatial and temporal resolution gap between light microscopy observations and EM, by enabling the overlay of fluorescence and electron microscopy images from the same cell. This allowed us to complement dynamic information from live cell imaging with high‐resolution ultrastructural information, providing unique data on the location of a fluorescently labeled molecule within the ultrastructural context of a cell^37^. We observed, first, a stereotypical evolution of an aggregate forming with a dense central core of high FRET intensity, surrounded by a rim of lower FRET intensity, throughout the neuronal soma and processes. Second, we observed that aggregation was heterogeneous in location, occurring in multiple hotspots throughout the cell body but often in crowded cellular environments rich in organelles, including membrane surfaces from the nucleus, plasma membrane, Golgi and mitochondria. We then performed 3D CLEM using a mitochondrial marker to align the mitochondria in fluorescence and electron microscopy. This overlay revealed clear localization of the donor A53T a-Syn monomer to the mitochondria. The increase in aggregation kinetics of α-Syn inside the cell was related to multi-focal seeding events occurring at different membrane surfaces, accelerating primary and secondary nucleation processes. Thus, multiple seeding events throughout the human neuron may occur, involving key organelles such as the mitochondria.

Mitochondrial dysfunction and synucleinopathy interact in the pathogenesis of PD^38,39^. α-Syn has been shown to selectively bind to mitochondrial membranes^12,40–42^ and may play a physiological role in mitochondrial function, mediating maintenance of mitochondrial fission, complex I activity and calcium signaling (reviewed in refs. ^43,44^). In in vivo models, overexpression of human α-Syn A53T, specifically in dopaminergic neurons, led to the formation of mitochondrial inclusions, which preceded neuronal loss^45^. Mitochondrial membranes are made up of a range of different phospholipids, although CLs are specific phospholipids of the mitochondria, comprising about 20% of the inner mitochondrial membrane phospholipids mass, where it maintains the structural and functional integrity of proteins involved in the mitochondrial function^46^. Previous work has provided key observations, namely that (1) in vitro, α-Syn directly binds^47^ CL and fragments CL-containing artificial membranes; and (2) SNCA-mutant neurons display fragmented mitochondria, and CL translocation to the outer mitochondrial membrane (OMM) in A53T SNCA-mutant neurons binds mutant α-Syn and facilitates the folding of α-Syn to an α-helix^23^.

Here, we reveal two key mechanisms by which mitochondria seeds the aggregation of α-Syn. We show that (1) A53T α-Syn binds CL inside and outside cells; (2) CL is a potent trigger for the rapid aggregation of A53T α-Syn to form fibrils, without a lag phase in vitro; and (3) during CL-induced aggregation, the CL is sequestered within the protein aggregate as it forms, and this lipid–protein co-assembly could act as a reactant for further surface-induced elongation. Previous studies using nuclear magnetic resonance and cryo-EM highlight how lipids co-assemble with α-Syn molecules, implying strong lipid–protein interactions^48^. These findings contribute to the growing understanding of the biogenesis of an LB. LBs have been shown to have complex compositions and organizations: they contain fibrillar α-Syn as well as non-proteinaceous material (lipids) and membranous organelles^49^. Previous work^50^ adopting CLEM imaging of inclusions generated by seeding with pre-formed fibrils shows enrichment of lipids that are specific for mitochondria, endoplasmic reticulum and Golgi apparatus membranes. Our work suggests that, in the absence of fibrillar seeds, the lipid membranes within the cell act as the seed for aggregation, and, critically, this can be achieved by several membrane surfaces, including CL in mitochondria. Self-assembly and primary nucleation starts at a (CL-containing) membrane surface and sequesters the lipid within it as the aggregate grows into a mature structure.

In addition to providing the lipid seed for aggregation, mitochondria are a critical source of ROS. A53T α-Syn inhibits complex I–dependent respiration, impairs ATP production and depolarizes the mitochondrial membrane, leading to an increase in generation of ROS. The oxidative environment, in turn, promotes further oligomerization of A53T monomers in neurons. Inhibition of mROS formation can suppress A53T oligomerization and abolish A53T-induced toxicity. Therefore, inside cells, the effect of mROS is bi-directional and self-amplifying: mROS promotes oligomerization of α-Syn, and oligomer formation impairs complex I function and induces further mROS generation, driving the aggregation reaction.

The final common pathway of A53T within the mitochondria is the altered permeability of the OMM. We previously reported that WT α-Syn oligomers of the Type-B structure are located at the mitochondrial membrane and can generate the production of free radicals, which induce local oxidation events in mitochondrial lipids, resulting in mitochondrial lipid and protein peroxidation; together, these events lead to mitochondrial membrane permeabilization^12^. In this study, we show that the application of A53T monomers to isolated mitochondria or whole cells induces rapid permeability transition of the mitochondrial membrane, similarly to the effect of the WT α-Syn Type-B oligomer. These data, taken together with the CL-induced aggregation, raise the hypothesis that A53T contacts CL, triggering oligomer formation and conversion to Type-B oligomers. This results in oligomer-induced impaired respiratory chain function and reduced ATP production, oligomer-induced mROS production and oligomer-induced mitochondrial membrane depolarization. Together, these effects predispose mitochondria to the early opening of the PTP and activation of caspase 3–dependent apoptosis.

hiPSC-derived neurons bearing SNCA-A53T mutation model the effect of the aggregation of endogenous α-Syn in disease^51–53^. In agreement with our findings that A53T α-Syn exhibits increased oligomerization, we identified higher levels of oligomers in the SNCA-A53T neurons. Increased aggregation in SNCA-A53T was associated with impaired respiration, depolarized mitochondrial membrane potential and oxidative stress. The A53T cellular environment, with intracellular aggregates and oxidative stress together, resulted in accelerated oligomerization of exogenously added α-Syn. Finally, the presence of A53T oligomers was sufficient to induce early opening of the mPTP and result in cell death. Removal of one of the major drivers of mPTP and oligomerization, mROS, was able to prevent the cellular pathology in the SNCA-A53T model, thereby supporting our hypothesis that the mitochondria is a key mediator of α-Syn pathology.

## Methods

### Human recombinant α-Syn

#### Aggregation of human recombinant α-Syn

Monomeric WT and mutant (A53T, A30P and E46K) α-Syn was purified from *Escherichia coli*, as previously described in ref. ^55^. Monomeric WT and mutant (A53T, A30P and E46K) α-Syn was labeled with either maleimide-modified AF488 or AF594 dyes (Invitrogen) via the cysteine thiol moiety, as previously reported^56^. The effect of the tag on the aggregation kinetics, and the kinetics of mutant-labeled α-Syn, have been described previously^10^. The protocol for generating α-Syn oligomers is similar to that described previously^18^. In brief, a 70 μM solution made up of equimolar concentrations of AF488-labeled and AF594-labeled α-Syn was prepared in 25 mM Tris-HCl (pH 7.4) and 100 mM NaCl. The buffer was freshly prepared before each experiment and passed through a 0.02-μm syringe filter (Anotop, Whatman) to remove insoluble contaminants. The aggregation mixture was incubated at 37 °C with orbital shaking at 200 r.p.m., and timepoints were taken at 0 hours, 24 hours and 7 days.

### Cell culture

Primary rat cortical co-culture is described in the Supplementary Methods. hiPSCs were derived from donors who had given signed informed consent for the derivation of hiPSC lines from skin biopsies as part of the European Union Innovative Medicines Initiative–funded program StemBANCC and reprogrammed. Controls 1 and 2 were derived by StemBANCC from an unaffected volunteer, and control 3 was purchased from Thermo Fisher Scientific. hiPSCs were generated from familial patients with PD carrying a point mutation in *SNCA* (A53T point mutation). The presence of mutations was confirmed by a Sanger sequence (GENEWIZ). The iso-CTRL line of *SNCA* A53T was generated using CRISPR–Cas9 editing by Applied StemCell (project ID: C1729). Neural induction and differentiation were performed using a modified, published protocol^57^.

### Immunohistochemistry

Standard immunocytochemistry was performed for the antibodies listed in Supplementary Table 5. For aptamer staining, see the Supplementary Methods.

### Live cell imaging

Live cell imaging was performed using an epi-fluorescence inverted microscope equipped with a CCD camera (Retiga, QImaging) or confocal microscope (Zeiss LSM 710 or 880 with an integrated META detection system).

Superoxide production: Cells were washed and loaded with 2 uM dihydroethidium (HEt, Thermo Fisher Scientific) in the recording buffer. The ratio of the fluorescence intensity, resulting from its oxidized/reduced forms, was quantified, and the rate of ROS production was determined by dividing the gradient of the HEt ratio after application of recombinant α-Syn against the basal gradient. mROS was assessed using MitoTracker Red CM-H2XRos dye (Thermo Fisher Scientific)—a reduced non-fluorescent version of MitoTracker Red that is fluorescent upon oxidation within mitochondria, which accumulates in mitochondria upon oxidation. The rate of increase in red fluorescence for each cell was analyzed as the production of mROS. Mitochondrial membrane potential (Δψ_m_): Cells were loaded with 25 nM TMRM (Thermo Fisher Scientific), a lipophilic cationic dye that accumulates within mitochondria in inverse proportion to Δψ_m_ according to the Nernst equation, in the recording buffer for 40 minjtes at room temperature. [Ca^2+^]c imaging was performed using Fura-2 AM, which is a ratiometric dye with a high affinity for Ca^2+^, or the Fluo-4 AM calcium indicator. NADH autofluorescence was measured as described in refs. ^58,59^ using an epi-fluorescence inverted microscope with excitation at a wavelength of 360 nm within 455-nm emission. The total NADH pool was calculated by subtracting the minimal autofluorescence (by 1 μM FCCP) from the maximal signal (by 1 mM NaCN). The NADH redox state was expressed as the ratio between the maximal and minimal reduction. ATP was measured by transfecting cells with the mitochondrial-targeted ATP indicator AT1.03 (ref. ^60^), allowing visualization of the dynamics of ATP. The ratio between yellow fluorescence protein (YFP) and cyan fluorescence protein (CFP) was measured after the application of each monomer sample. mPTP opening: Cells were loaded with 25 nM TMRM and either 5 μM NucView 488 (Biotium) or 5 μM Fluo-4 AM (Thermo Fisher Scientific). The threshold of mPTP opening was measured as the timepoint at which rapid loss of TMRM fluorescence occurred after applying ferutinin or α-Syn samples. Cell death assay: Cell death was detected using propidium iodide (PI, Thermo Fisher Scientific) or SYTOX Green (SYTOX, Thermo Fisher Scientific) and Hoechst 33342 (Hoechst, Thermo Fisher Scientific) to count the total number of cells. Detection of aggregates: Aggregates were detected using a fluorescent amyloidogenic marker, Amytracker 540 (Ebba Biotech). Visualization of CL in cells: CL inside cells was detected using 10-*N*-NAO, a fluorescent reporter of CL (Biotium^61^).

Intracellular FRET measurement: AF488-labeled and AF594-labeled α-Syn was applied to cells cultured in 8-ibidi chambers (cat. no. IB-80826). After a range of incubation times, cells were washed twice and replaced with HBSS. Controls including either AF488-only or AF594-only labeled treated cells were also measured under the same conditions. As a measure of total α-Syn, the AF594-labeled α-Syn was directly excited, and its intensity was measured (the AF488 was not used for this, because its intensity is also affected by the FRET process). As a measure of aggregated species, AF488 was excited by 488-nm irradiation, and emission was detected from AF594 (584–660 nm).

Intracellular FRET efficiency was calculated using a custom-written script in Igor Pro (WaveMetrics). In brief, after subtraction of the autofluorescence, the images were thresholded using the directly excited AF594 channel. The FRET efficiency for each voxel containing AF594 intensity was then calculated according to Equations 1–3 (refs. ^18,62^).

1 $$E = \frac{{I_A}}{{I_A + I_D}}$$

where *I*_*D*_ and *I*_*A*_ are the modified intensities in the donor and acceptor channels with donor excitation only:

2 $$I_D = D - A_D$$

3 $$I_A = A - A_A - C \times D$$

where *D* and *A* are the intensities from the donor and acceptor channels with donor excitation only, respectively; A_D_ and A_A_ are the instrument-specific autofluorescence in the donor and acceptor channels, measured in the absence of fluorophores; and C is the instrument-specific cross-talk from the donor to acceptor channel. The cross-talk from acceptor to donor channel is negligible.

To determine structural conversion (Fig. 1f), the FRET efficiencies from each timepoint and each α-Syn variant (WT, A53T, A30P and E46K) were binned into histograms (bin width = 0.05) and were globally fit to two Gaussian distributions (Igor Pro, WaveMetrics) (shared x-center and x-width). These were then integrated, and the fraction converted was defined as the area under the higher FRET efficiency peak divided by the total area. To determine the effect of the fitting error on the overall calculation of the populations, the errors were propagated for one dataset and are represented in Extended Data Figs. 2d and 3a.

To analyze the effects of different treatments on α-Syn oligomerization, we presented the mean intracellular FRET efficiency for each cell (Fig. 6diii). To estimate the CIs, we bootstrapped the single-cell data (using scipy.stats.bootstrap (version 1.7.3) in Python 3.9 using 9,999 resamples).

### Single-molecule confocal microscopy

Cells were treated with AF488-labeled and AF594-labeled monomers and incubated for various timepoints. Then, the lysates were collected using a lysis buffer (150 mM sodium chloride, 1% Triton X and 50 mM Tris (pH 8.0)) and analyzed using single-molecule confocal microscopy^18^. The samples were first diluted to concentrations ~50 pM before being loaded into a 200-μl gel-loading tip (Life Technologies) attached to the inlet port of a microfluidic channel (25 μm in height, 100 μm in width and 1 cm in length) mounted onto the single-molecule confocal microscope. The confocal volume was focused 10 μm into the center of the channel, and the solution was passed through the channel at an average velocity of 2 cm s^−1^ by applying a negative pressure, which was generated using a syringe pump attached to the outlet port via Fine Bore Polyethylene Tubing (0.38 mm inner diameter, 1.09 mm outer diameter; Smiths Medical). After the appearance of single-molecule bursts corresponding to labeled α-Syn passing through the confocal volume, the sample was measured for 600 seconds.

The confocal microscope is similar to those used previously^18^ (Supplementary Methods). The data were analyzed using custom-written scripts written in Igor Pro (WaveMetrics). Coincident events were those that had at least 10 photon counts bin^−1^ in each channel. After accounting for autofluorescence and cross-talk (Equations 1–3), the FRET efficiency (Equation 1) and approximate size of each oligomer (Equation 4) were calculated from the intensities in each channel:

4 $${\mathrm{Approximate}}\,{\mathrm{size}} = \frac{{2\left( {I_D + \frac{1}{\gamma }I_A} \right)}}{{I_{\mathrm{monomer}}}}$$

where γ is the experimentally determined gamma factor, corresponding to the relative detection efficiencies of the two dyes by the instrumentation and their quantum yields, and *I*_monomer_ is the average monomer brightness, calculated from the average of the non-coincident donor channel bursts intensities. As described in ref. ^10^, the size was estimated using the total fluorescence intensity and equated to the number of AF488 fluorophores present (because this is the only fluorophore directly excited). The term ‘apparent size’ is used because it is not possible to accurately determine sizes with high accuracy using single-molecule confocal microscopy (due to the stochastic nature of excitation and emission and the varied paths that the molecules can take through the excitation volume).

Oligomers were divided into three groups depending on their approximate size, as previously published^17,18^. The smallest oligomers we detect correspond to apparent dimers, and the remaining oligomers are classified as medium in size (up to 20 monomer units per oligomer). The division into Type-A and Type-B oligomers was made by globally fitting the medium-sized oligomer FRET efficiency histograms (shared x-center and x-width for all timepoints and treatments) to two Gaussian distributions (Type-A has a lower FRET efficiency than Type-B). The global fitting ensures that the same populations are identified from each dataset. To determine the effect of the fitting error on the overall calculation of the populations, the errors were propagated for one dataset and are represented in Extended Data Figs. 2d and 3a.

The fraction of coincidence was calculated by dividing the number of coincident events by the total number of events having counts above 10 photons bin^−1^ in the AF488 channel. For comparison, for a dual-labeled DNA sample in which ~100% of the molecules should be able to undergo FRET, the coincidence is ~0.10 under these experimental conditions^62^.

### Total internal reflection fluorescence microscopy: labeled α-Syn imaging

The dual-labeled α-Syn samples were diluted to nanomolar concentrations to facilitate single-molecule imaging^63^. Samples were then added to a cover slide on the inside of the chamber and incubated for at least 30 minutes before being washed with 20-nm filtered buffer (20 mM Tris and 100 mM NaCl, pH 7.1). Imaging was performed using a home-built, bespoke single-molecule TIRF microscope, as described previously^64,65^. Fluorophore-labeled samples were illuminated with a 488-nm laser (∼50 W cm^–2^), and the intensity of the emission from AF488 was measured over a time period of 10 seconds and recorded as a TIFF stack. Only those species that also had intensity resulting from AF594 due to FRET were analyzed. All image processing was performed using ImageJ.

### Single-molecule localization microscopy in cells

iPSC-derived cortical neurons were grown on glass-coated ibidi chambers, fixed and permeabilized, followed by non-specific blocking for 2 hours at room temperature. The samples were then incubated with 100 nM of the aptamer, and neurons were washed and incubated with a primary Tomm20 antibody (Santa Cruz Biotechnology, sc-17764). Single-molecule localization microscopy (SMLM) was performed on a Nanoimager super-resolution microscope (Oxford Nanoimaging) equipped with an Olympus 1.4 NA ×100 oil immersion super apochromatic objective. SMLM performed on the Nanoimager was partly analyzed using the Oxford Nanoimaging–developed online software, CODI.

### CLEM

Cells were plated and grown in 35-mm gridded glass-bottom dishes (P35G-1.5-14-CGRD, MatTek). Cells were fixed and then washed in 0.1 M PB, and confocal images using Zeiss LSM 880 (×63/1.4 oil DIC UV-VIS-IR M27 objective) were acquired from the area of interest (LD LCI Plan-Apochromat ×25/0.8 Imm Korr DIC M27 objective for tiling scan). Samples were then post-fixed in 2.5% glutaraldehyde/4% formaldehyde in 0.1 M PB (pH 7.4) for 30 minutes and stained in 1% reduced osmium (1% osmium/1.5% potassium ferricyanide) at 4 °C for 1 hour, followed by 1% tannic acid for 45 minutes at room temperature, before quenching in 1% sodium sulfate at room temperature for 5 minutes and washing in double-distilled water (3 ×5 minutes). The glass coverslip was dehydrated, embedded in Durcupan (44610-1EA, Sigma-Aldrich) and polymerized at 65 °C for 48 hours. The glass coverslip was removed from the resin by submerging in liquid nitrogen, and the cells of interest were relocated using the alphanumeric grid imprinted on the surface of the resin block. The block was trimmed to the cell of interest and serial sectioned at a thickness of 70 nm using a UC7 ultramicrotome (Leica Microsystems) and a diamond knife (Diatome) and collected onto 2 ×1-mm copper slot grids with a formvar support film (G089, TAAB Laboratories Equipment). Sections were post-stained using Reynold’s lead citrate and 1% uranyl acetate. Serial images of the cell were then acquired using a transmission electron microscope (Tecnai Spirit BioTwin, Thermo Fisher Scientific) operated at 120 keV.

### Correlative light and focused ion beam scanning electron microscopy

Cells were cultured, fixed and imaged using confocal microscopy as described above. For post-fixation and staining, a modified version of the National Center for Microscopy and Imaging Research method was used^66^. Cells were post-fixed in 2.5% glutaraldehyde/4% formaldehyde in 0.1 M PB (pH 7.4) at room temperature for 30 minutes and stained in 1% reduced osmium (1% osmium/1.5% potassium ferricyanide) at 4 °C for 1 hour. Cells were then treated with 1% thiocarbohydrazide (TCH) for 20 minutes at room temperature, followed by 2% osmium tetroxide for 30 minutes at room temperature and incubated overnight at 4 °C in 1% uranyl acetate. The next day, cells were stained en bloc with lead aspartate (pH 5.5) at 60 °C for 30 minutes. The glass coverslip was then removed from the MatTek dish and underwent graded dehydration in ethanol (25%, 50%, 70%, 90% and 100%, 5 minutes per step), followed by embedding in Durcupan and polymerization at 65 °C for 48 hours. The coverslips were subsequently removed from the resin block using liquid nitrogen, and the cells were relocated using grid coordinates. Areas of 3 × 3 grid squares in size with the cell of interest located at the center were then trimmed out using a razor blade, removed from the resin block^67^ and mounted on a 12.7-mm SEM pin stub (10-002012-100, Labtech) using silver paint (AGG3691, Agar Scientific). After mounting, each sample was sputter coated with a 10-nm layer of platinum (Q150S, Quorum Technologies). Focused ion beam scanning electron microscopy (FIB SEM) was carried out using a Crossbeam 540 FIB SEM with Atlas 5 for 3D tomography acquisition (Zeiss).

### Dynamic light scattering

Liposomes were prepared (Supplementary Methods), and the size distribution of lipid vesicles was determined by an ALV/CGS-3 platform-based goniometer system (ALV). This instrument was equipped with a 22-mW HeNe laser with a wavelength of 633 nm, and backscattered light was detected at an angle of 90° at room temperature. Lipid vesicle stock solutions were diluted to 150 µM in a filtered 25 mM phosphate buffer (pH 7.4), transferred into a glass test tube and placed in the measurement cell. Three measurements were performed for the sample for 30 seconds at room temperature, and average values were used for analyzing the data.

### Aggregation assay

ThT fluorescence time course measurements were performed in 96-well microliter plates using FLUOstar Omega microplate reader (BMG Labtech) with excitation and emission wavelengths set to 450 nm and 485 nm. Then, 50 µM protein samples were incubated in the presence and absence of lipid vesicles (400 µM) with 20 µM ThT in 25 mM Tris buffer (pH 7.4). Each sample well contained 100 µl of the reaction mixture, and spontaneous aggregation was induced by incubation at 37 °C without shaking. A minimum of three trials were performed to ensure its reproducibility.

### CD spectroscopy

A Jasco J-810 spectrometer was used for CD measurements. Samples containing 10 µM WT α-Syn alone and in the presence of CL (80 µM and 160 µM) were prepared in a 25 mM phosphate buffer (pH 7.4) and measured immediately after mixing. Experiments were performed at 25 °C using a quartz glass cell with 1-mm path length. The samples were recorded from 190 nm to 260 nm wavelength with a total of three scans for each sample.

### TEM

Selected fibril samples from 96-well plates were diluted to 3 µM in 25 mM phosphate buffer (pH 7.4). An aliquot (7 μl) of diluted sample was placed onto carbon support film 300 mesh, 3-mm copper grids (TAAB Laboratories Equipment), and allowed for 3 minutes and blot dried. The TEM grids were subsequently stained using 7 μl of 1% uranyl acetate for 3 minutes, followed by blot drying. Samples were imaged on a Thermo Fisher Scientific Tecnai F20 electron microscope (200 kV, field emission gun) equipped with an 8k × 8k CMOS camera (TVIPS F816).

### TIRF microscopy: CL imaging

Biotinylated liposomes were prepared as described previously^62^. Imaging was performed in a home-built total internal reflection microscope, as described previously^68^. Amyloid fibrils prepared in the presence of biotin-conjugated CL were incubated with 1 nM AF647-streptavidin (Thermo Fisher Scientific) and 5 mM ThT (Sigma-Aldrich) for 10 minutes at room temperature. Images were recorded for 50 frames from the red channel (AF647 emission) with 641-nm illumination, followed by the green channel (ThT emission) with 488-nm illumination. The images generated from AF647-streptavidin and SAVE imaging with ThT were analyzed using ImageJ and a custom-written script in Igor Pro (WaveMetrics). The percentage coincidence was determined from the number of coincident aggregates and the total number of aggregates detected. The mean and standard deviations were calculated across the image sets.

### Statistical analysis

Origin 2020 (MicroCal, https://www.originlab.com/2020; RRID: SCR_014212) software was employed for the statistical analysis and exponential curve fitting. An assessment of the normality of data was performed using Shapiro–Wilk and Kolmogorov–Smirnov normality tests. Statistical tests were performed using unpaired two-sample *t*-tests, one-way ANOVA or two-way ANOVA corrected with Tukey’s honest significant difference post hoc test. Experimental data are shown as data points ± s.e.m. or s.d., and *P* value was set at 0.05. Sample sizes for experiments were selected to capture technical variation, including numbers of cell/field of view and coverslips, and biological variation, including numbers of the animal batch for primary co-culture and independent inductions and clones or patient lines for hiPSC-derived neurons. Sample sizes were not predetermined but are similar to those reported in previous publications^12,69,70^. *F*-statistics were used to estimate variance within each group. Data collection was randomized, and the collection and analysis were performed blinded to the conditions of the experiments. No data points were excluded from the analyses. For live imaging experiments in human neurons, application of standard controls was used to determine the dynamic range of the signal measured (the maximum and minimum signal). The data are normalized to the maximum and minimum, and the same experimental conditions were used to compare two states—that is, control versus A53T mutant. In the absence of a known standard in these experiments, comparisons across different conditions and cell lines are relative only to each other within this experimental paradigm.

### Reporting summary

Further information on research design is available in the Nature Research Reporting Summary linked to this article.

## Online content

Any methods, additional references, Nature Research reporting summaries, source data, extended data, supplementary information, acknowledgements, peer review information; details of author contributions and competing interests; and statements of data and code availability are available at 10.1038/s41593-022-01140-3.

## Supplementary information

Supplementary Information Supplementary Methods and Supplementary Tables 2, 3 and 5

Reporting Summary

Supplementary Table 1 Detailed statistics for the main figures and the extended data

Supplementary Table 4 Details and the location of imaging and tabular datasets, codes and software used for this study

## Data Availability

Imaging and tabular datasets used in this study are available and have been deposited in Zenodo (locations are listed in Supplementary Table 4). Source data are provided with this paper.

## Source data

Source Data Fig. 1 Statistical source data for Fig. 1
Source Data Fig. 2 Statistical source data for Fig. 2
Source Data Fig. 4 Statistical source data for Fig. 4
Source Data Fig. 5 Statistical source data for Fig. 5
Source Data Fig. 6 Statistical source data for Fig. 6
Source Data Fig. 7 Statistical source data for Fig. 7
Source Data Extended Data Fig. 1 Statistical source data for Extended Data Fig. 1
Source Data Extended Data Fig. 2 Statistical source data for Extended Data Fig. 2
Source Data Extended Data Fig. 3 Statistical source data for Extended Data Fig. 3
Source Data Extended Data Fig. 4 Statistical source data for Extended Data Fig. 4
Source Data Extended Data Fig. 5 Statistical source data for Extended Data Fig. 5
Source Data Extended Data Fig. 7 Statistical source data for Extended Data Fig. 7
Source Data Extended Data Fig. 8 Statistical source data for Extended Data Fig. 9
Source Data Extended Data Fig. 10 Statistical source data for Extended Data Fig. 10

## Code availability

Code used in this study has been deposited in relevant repositories (Supplementary Table 4).
