# Author Correction: Pathological structural conversion of α-synuclein at the mitochondria induces neuronal toxicity

**DOI:** 10.1038/s41593-022-01206-2

**Published:** 2022-10-19

**Authors:** Minee L. Choi, Alexandre Chappard, Bhanu P. Singh, Catherine Maclachlan, Margarida Rodrigues, Evgeniya I. Fedotova, Alexey V. Berezhnov, Suman De, Christopher J. Peddie, Dilan Athauda, Gurvir S. Virdi, Weijia Zhang, James R. Evans, Anna I. Wernick, Zeinab Shadman Zanjani, Plamena R. Angelova, Noemi Esteras, Andrey Y. Vinokurov, Katie Morris, Kiani Jeacock, Laura Tosatto, Daniel Little, Paul Gissen, David J. Clarke, Tilo Kunath, Lucy Collinson, David Klenerman, Andrey Y. Abramov, Mathew H. Horrocks, Sonia Gandhi

**Affiliations:** 1 Department of Clinical and Movement Neurosciences, UCL Queen Square Institute of Neurology, London, UK; 2 The Francis Crick Institute, London, UK; 3 Aligning Science Across Parkinson’s (ASAP) Collaborative Research Network, Chevy Chase, MD USA; 4 EaStCHEM School of Chemistry, University of Edinburgh, Edinburgh, UK; 5 School of Physics, University of Edinburgh, Edinburgh, UK; 6 Department of Chemistry, University of Cambridge, Cambridge, UK; 7 Dementia Research institute at University of Cambridge, Cambridge, UK; 8 Institute of Cell Biophysics, Russian Academy of Sciences, Pushchino, Russia; 9 Cell Physiology and Pathology Laboratory, Orel State University, Orel, Russia; 10 Istituto di Biofisica, National Council of Research, Trento, Italy; 11 MRC Laboratory for Molecular Cell Biology, University College London, London, UK; 12 NIHR Great Ormond Street Hospital Biomedical Research Centre, London, UK; 13 Centre for Regenerative Medicine, Institute for Stem Cell Research, School of Biological Sciences, University of Edinburgh, Edinburgh, UK

**Keywords:** Parkinson's disease, Biophysics, Mitochondria

Correction to: *Nature Neuroscience* 10.1038/s41593-022-01140-3, published online 30 August 2022.

In the version of this article initially published, the acknowledgement of support from Aligning Science Across Parkinson’s did not include the grant no. ASAP-000509, and Supplementary Table 4 did not include linked materials and methods. The HTML and PDF versions of the article and the accompanying Supplementary material have now been updated.

