# Supplementary material for: Pathological structural conversion of α-synuclein at the mitochondria induces neuronal toxicity
Source: Nat Neurosci. 2022 Aug 30;25(9):1134–48. doi: 10.1038/s41593-022-01140-3 (PMC9448679; doi:10.1038/s41593-022-01140-3)
Supplement: Supplementary file 1 — Supplementary Methods and Supplementary Tables 2, 3 and 5 [file 41593_2022_1140_MOESM1_ESM.pdf]

nature portfolio

https://doi.org/10.1038/s41593-022-01140-3

Supplementary information

Pathological structural conversion of α-synuclein at the mitochondria induces neuronal toxicity

In the format provided by the authors and unedited

SI Method

Human recombinant α-Syn

Aggregation of human recombinant α-Syn

Monomeric WT and mutant (A53T, A30P and E46K) α-Syn was purified α-Syn was purified α-Syn was purified from *Escherichia coli* as previously described in [1]. Aggregation reactions were carried out using a solution of α-Syn 70 µM in 25 mM Tris buffer supplemented with 100 mM NaCl, pH 7.4 (in the presence of 0.01% NaN_3 to prevent bacterial growth). The buffer was freshly prepared before each experiment and passed through a 0.02 µm syringe filter (Anotop, Whatman) to remove insoluble contaminants. Prior to incubation, the reaction mixture was ultra-centrifuged at 90k x g. for 1h at 4 °C to remove potential seeds. The supernatant was collected and separated in two fractions: one kept at 4°C at all times until use (monomers), and a second incubated in the dark at 37 °C and 200 r.p.m., during 7-8 hours to avoid fibril formation (monomers + oligomers). α-Syn was always kept in LoBind microcentrifuge tubes (Eppendorf, Hamburg, Germany) to limit surface adsorption. For experiments testing the effect of the presence of endogenous protein on *in vitro* A53T aggregation, 7 µM unlabeled recombinant WT α-Syn was added at the start of the aggregation procedure.

Labeling of α-Syn WT, A53T, A30P and E46K

Monomeric WT and mutant (A53T, A30P and E46K) α-Syn was labelled with either maleimide-modified AF488 or AF594 dyes (Invitrogen, Carlsbad, CA, USA) via the cysteine thiol moiety as previously reported [2]. The labeled protein was purified from the excess of free dye by a P10 desalting column with Sephadex G25 matrix (GE Healthcare, Waukesha, WI, USA), divided into aliquots, flash frozen, and stored at -80 °C [3]. Each aliquot was thawed immediately and used only once. The reaction yield was checked by mass spectrometry for all reactions, and all labelling reactions with a yield lower than 90% were discarded.

Throughout this work, non-acetylated a-Syn is used. Whilst N terminal acetylated α-Syn may be physiologically relevant, the effect of acetylation on aggregation kinetics remains unclear. Some studies show an absence of impact on fibrillation [4], while others showing slower fibril growth rates for the acetylated protein [5, 6]. This may also depend on which α-Syn mutations are being observed, with A53T having shown to have slower fibrillation rates while acetylated compared to non-acetylated A53T [5], with oligomerization of other familial mutants also shown to be affected by acetylation [7]. Notably acetylated protein seems to present similarly with regards fibril morphologies [5, 6]. Non-acetylated α-Syn was selected from monomeric WT α-Syn purified from *Escherichia coli* [1].

Cell culture

Primary cell culture

Primary rat cortical co-culture, derived from 1-3 days postpartum Sprague Dawley rats (University College London breeding colony) were used and experimental procedures were performed according to the United Kingdom Animal (Scientific Procedures) Act of 1986. Following cortical dissection, digestion and dissociation, the cultures were maintained at 37 °C (5% CO2) and neurons were used at 12-16 days.

Rat cortices were placed in an ice-cold dissecting buffer (HBSS supplemented with 10 mM HEPES and 20% FBS) and washed five times with a washing buffer (HBSS supplemented with 10mM HEPES). The tissue was digested with 0.5% EDTA-trypsin supplemented with DNAse for 15 min and neutralized with the dissecting buffer. After washing twice again with the washing buffer, the tissues were dissociated with washing buffer supplemented with DNAse, and then the pellets were collected in Neurobasal completed medium (Neurobasal A medium supplemented with B27, 2 mM Glutamax, Pen/Strep). Approximately 600,000 cells were plated on 25 mm coverslips (PDL coated) and 200,000 cells for 8-well ibidi chambers (PDL coated). The cultures were maintained at 37 °C (5% CO2), and the media was changed every 4-5 days. Cells were used at 12-16 days.

iPSC culture

Briefly, the CytoTune-iPS reprogramming kit (ThermoFisherScientific) was used to reprogram fibroblasts through expression of OCT4, SOX2, KLF4 and c-MYC by four separate Sendai viral vectors. The isogenic series of iPSC lines with a range of SNCA alleles (SNCA null, SNCA 2 alleles and SNCA 4 alleles) were generated as described in [8] The Isogenic control line of *SNCA* A53T was generated using CRISPR/Cas9 editing by Applied StemCell *Inc*. (USA, project ID: C1729). Briefly, SNCA A53T repair primer was designed based on the sequence indicated above with 30 bps at both ends at 5’ and 3’ arms. A restriction enzyme site (Sfcl) was created once repair primer is introduced so that PCR product digested by Sfcl with T53A gene-corrected lines will have two sizes of products (250bps and 265 bps) while the mutant line will have only one 520bp product. Transfection was performed by Lipofectamine 2000, and cells were sorted on the next day. Sorted cells were cultured, and single colonies were picked for initial PCR screening. Potential gene-corrected clones were further confirmed by sanger sequencing. Based on the Sanger sequencing result and cell growth status, clones were chosen for further expansion and cryopreserved into 5 vials. One random vial from each clone was used for final QC analysis. gDNA samples of both clones were also checked for two off-target sites.

hiPSCs were maintained on Geltrex in Essential 8 medium or mTeSR (ThermoFisherScientific) and passaged using 0.5 mM EDTA. All lines were mycoplasma tested (all negative) and performed with short tandem repeat profiling (all matched) by the Francis Crick Institute Cell service team.

Differentiation into Cortical Neurons

Briefly, upon 100% confluence of hiPSC, dual SMAD inhibitors SB431542 (10 µM, Tocris) and Dorsomorphin (1 µM, Tocris), were added to N2B27 medium (DMEM:F12, insulin, 2-mercaptoethanol, Non Essential Amion Acids, N2 supplement, Pen/Strep, Neurobasal, B27, Glutamax, Pen/Strep) and the medium was refreshed every day for 10-12 days until

neuroepithelium appear. Then the neuroepithelium sheets were split using dispase and cultured in N2B27. At around 35 days of induction, cells were dissociated into a single cell using accutase and approximately 150,000 number of cells plated on either PDL and laminin coated glass bottom 8-well slide chambers (Ibidi/Thistle, cat No.80826), Geltrex-coated 8-well ibidi chambers (cat No. IB-80826) or 96-well plates (Falcon, cat No. 353219). The medium was replaced every 4-5 days and cells were used at 60-90 days after induction.

Live cell Imaging

Live-cell imaging was performed using an epi-fluorescence inverted microscope equipped with a CCD camera (Retiga; QImaging) or confocal microscope (Zeiss LSM 710 or 880 with an integrated META detection system). For epi-fluorescence inverted microscope, excitation was provided by a xenon arc lamp with the beam passing through a monochromator (Cairn Research) and emission was reflected through a long-pass filter to a cooled CCD camera and digitized to 12-bit resolution (Digital Pixel Ltd, UK). The data were analyzed using Andor iQ software (Belfast, UK). For confocal microscopes, illumination intensity was limited to 0.1-0.2% of laser output to prevent phototoxicity, and the pinhole was set to allow optical slice at approximately 1-2 µm. Room temperature (RT) HBSS was used as a recording buffer. 3-6 fields of view per well and at least 3 wells per group were used to analyze using ZEISS ZEN software, Volocity 6.3 cellular imaging or ImageJ software. All experiments were repeated at least 2-3 times with different animal batches (primary co-culture) and inductions (hiPSC derived neurons).

Superoxide production:

HEt is an indicator of superoxide which exhibits blue fluorescence in the cytosol before oxidation, and the nucleus presents a red fluorescence upon oxidation. HEt allows the rate of superoxide generation to be measured which is present as the ratio of the oxidized form of the dye over the reduced form. The recording was performed using an epi-fluorescence inverted microscope equipped with 20x objective after a quick loading (2-3 min) in order to limit the intracellular accumulation of oxidized product, and the dye was present throughout the imaging. Excitation was at530 nm, and emission recorded above 560 nm was assigned to the oxidized form, while excitation at 380 nm and emission collected from 405 nm to 470 nm was assigned to the reduced form. The ratio of the fluorescence intensity, resulting from its oxidized/reduced forms, was quantified and the rate of ROS production was determined by dividing the gradient of the HEt ratio after application of recombinant α-Syn against the basal gradient.

Mitochondrial ROS

Cells were washed twice and loaded with 500 nM MitoTracker® Red CM-H2XRos for 20-30 min with the dye in the recording buffer at RT. The fluorescence measurement was obtained by using a confocal microscope with a 40x oil-immersion objective lens excited with 561 nm laser and emission detected above 580 nm. The rate of increase in red fluorescence for each

cell was analyzed as the production of mitochondrial ROS. The basal MitoTracker® Red CM-H2XRos signal prior to application of α-Syn is used as the comparison to the MitoTracker® Red CM-H2XRos signal post α-Syn treatment.

Mitochondrial Membrane Potential (ΔΨm)

ΔΨm is the electrogenic potential between the inner membrane and matrix of mitochondria which, in combination with the mitochondrial pH gradient, provides the force to drive protons into mitochondria to generate ATP. Cells were washed twice and loaded with 25 nM tetramethylrhodamine methyl ester (TMRM, Thermo Fisher Scientific), a lipophilic cationic dye that accumulates within mitochondria in inverse proportion to $\Delta\psi_m$ according to the Nernst equation, in the recording buffer for 40 min at RT. Confocal microscope imaging was performed in the presence of TMRM; the 560 nm laser was used to excite, and fluorescence was measured above 580 nm. Z-stack images were collected, and the fluorescence intensity of TMRM was analyzed using ZEISS ZEN software (Zeiss). The assessment of the maintenance of $\Delta\psi_m$was obtained on a single focal plane (time series) by application of 2 µg/ml Oligomycin (inhibitor of Complex V), 5 µM Rotenone (inhibitor of Complex I) and 1 µM FCCP (mitochondrial uncoupler). For analysis, mitochondria within a cell were identified by TMRM staining, and the mean intensity of TMRM signal was measured using a fixed threshold. 1 µM FCCP was applied at the end of the experiment to acquire the minimum value of fully depolarized $\Delta\psi_m$. Data is normalized to 100% (basal TMRM) and 0% (following addition of FCCP).

$[Ca^{2+}]c$ imaging

The cytosolic Ca^{2+} as well as the rapid transient kinetics and decay times were assessed. 5 uM Fura-2 or Fluo 4 was loaded with 1.25 uM Pluronic acid for 40 min and then washed twice before imaging. For the cells loaded with Fura-2, the fluorescence measurement was obtained on an epifluorescence inverted microscope equipped with a 20x objective. $[Ca^{2+}]c$ was monitored in a single cell by obtaining the ratio between the excitation at 340 nm (high Ca^{2+}) and 380 nm (low Ca^{2+}) for which fluorescence light was reflected through a 515 nm long pass filter. For the cells loaded with Fluo 4, confocal microscope imaging was performed after two washes. The 488 nm laser was used for excitation, and emission fluorescence above 519 was measured.

NADH redox state and total NADH pool

NADH redox state indicates the balance between electron transport chain (ETC) activity and the rate of substrate supply as a key mechanism to maintain functional mitochondria and energy production. 1 µM FCCP stimulates maximal respiration by completely oxidizing all mitochondrial NADH into NAD+ and H+ which results in the minimal NADH autofluorescence value (set at 0%). Following stabilization of the signal, 1 mM NaCN was added which inhibited the respiration, preventing NADH oxidation, resulting in the maximal NADH autofluorescence

value (set at 100%). The total NADH pool was calculated by subtracting the minimal autofluorescence (by 1 µM FCCP) from the maximal signal (by 1 mM NaCN). The NADH redox state was expressed as the ratio between the maximal and minimal reduction. Higher redox state indicates more reduced NADH which suggests Complex I inhibition [9, 10].

ATP is measured by transfecting cells with the mitochondrial-targeted ATP indicator AT1.03 (Imamura et al., 2009), allowing visualization of the dynamics of ATP. The ratio between yellow fluorescence protein (YFP) and cyan fluorescence protein (CFP) was measured after the application of each monomer sample. When ATP is not bound to the probe, a low FRET signal is detected, which is emitted from the CFP. However, upon binding of ATP to the probe, the two fluorescent proteins are close enough to generate a FRET signal which results in a YFP signal. Cells were excited with a 405 nm laser, and emission for CFP is at 460-510 nm and 540-600 nm for YFP.

mPTP opening:

The mPTP experiments were performed as previously described [11]. Whole cells were loaded with TMRM and the cytosolic calcium dye Fluo4, followed by stepwise application of the electrogenic calcium ionophore Ferutinin, a known inducer of mPTP opening by mitochondrial calcium overload [12](reviewed here [13]). Briefly, cells were washed twice and loaded with 25 nM TMRM and either 5 µM Nucview 488 (Biotium) or 5 µM Fluo4-AM (Thermo Fisher Scientific). The fluorescent signals were measured with a 488 nm laser for Nucview 488 and Fluo4 and emission between 488 nm and 516 nm. For TMRM, a 560 nm laser was used for excitation, and emissions were read above 580 nm after application of 30 µM Ferutinin at once or 2 µM Ferutinin in a stepwise manner that leads to mPTP opening upon reaching the threshold. The threshold of mPTP opening was measured as the time point at which rapid loss of TMRM fluorescence occurred after applying Ferutinin or α-Syn samples. We examined the relationship between mPTP opening and cell toxicity by co-loading TMRM and Nucview488 (an apoptotic marker of caspase-3 cleavage) and applying a high concentration of Ferutinin (30 µM) to open the mPTP. Opening the mPTP was then followed by an increase in nuclear green fluorescence, demonstrating caspase 3 cleavage and caspase 3-dependent apoptosis.

To permeabilize cells, cells were exposed to 40-60 µM digitonin in a pseudo-intracellular solution consisting of 135 mM KCL, 10 mM NaCl, 20 mM HEPES, 5 mM pyruvate, 5 mM malate, 0.5 mM KH_2PO_4, 1 mM $MgCl_2$, 5 mM EGTA, and 1.86 mM $CaCl_2$ yielding approximately 100 nM [Ca^{2+}]. 1µM FCCP was applied at the end of the experiment to confirm the mitochondria were maximally depolarised at PTP opening.

Cell death assay

Cell death was detected using Propidium iodide (PI, Thermo Fisher Scientific) or SYTOX™ Green (SYTOX, Thermo Fisher Scientific) which is excluded from viable cells but exhibits red fluorescence following a loss of membrane integrity and Hoechst 33342 (Hoechst, Thermo Fisher Scientific) which stains chromatin blue in all cells to count the total number of cells. 20 µM PI or 500 nM SYTOX and 10uM Hoechst were directly added into the dishes and cells were incubated for 15 min. The fluorescence measurements were detected using confocal

microscopy. Hoechst and PI were excited by 405 nm and 543 nm laser lines with the emission between 405 nm to 470 nm and 570 nm to 640nmrespectively. SYTOX was excited by a 488 nm laser with emission between 488 nm and 516 nm. The percentage of cell death was quantified by the number of red (PI) or green (SYTOX) fluorescent cells divided by the total number of Hoechst 33342 expressing cells per image. This data reflects the proportion of cells measured, and the ratio is compared across experimental conditions.

Detection of aggregates

Aggregates were detected using a fluorescent amyloidogenic marker, Amytracker™ 540 (Ebba Biotech). Briefly, cells were washed twice and loaded with 1% Amytracker for 20 min. Confocal microscope imaging was performed in the presence of Amytracker. The 560 nm laser was used to excite, and fluorescence emission above 580 nm was measured.

Visualization of cardiolipin in cells

Cardiolipin inside cells were detected using 10-N-Nonyl acridine orange (NAO), a fluorescent reporter of cardiolipin (Biotium, [14]). Cells were washed twice and loaded 1µM NAO for 20 min, and confocal microscope imaging was performed in the presence of NAO. The 488 nm laser was used to excite, and fluorescence above 519 was measured.

Single-molecule confocal microscopy

Cells were treated with AF488 and AF594 labelled monomers and incubated for various time points. Then the lysates were collected using a lysis buffer (150 mM sodium chloride, 1% Triton-X, 50 mM Tris pH 8.0) and analyzed using single-molecule confocal microscopy [15]. The samples were first diluted to concentrations ~50 pM before being loaded into a 200 µL gel-loading tip (Life Technologies, Carlsbad, CA, USA) attached to the inlet port of a microfluidic channel (25 µm in height, 100 µm in width, 1 cm in length) mounted onto the single-molecule confocal microscope (described below). The confocal volume was focused 10 µm into the center of the channel, and the solution was passed through the channel at an average velocity of 2 cm/s by applying a negative pressure, which was generated using a syringe pump attached to the outlet port via Fine Bore Polyethylene Tubing (0.38 mm inner-diameter, 1.09 mm outer diameter; Smiths Medical International, Hythe, Kent, UK). After the appearance of single-molecule bursts corresponding to labelled α-Syn passing through the confocal volume, the sample was measured for 600s.

The confocal microscope is similar to those used previously [15]. A Gaussian laser beam at 488 nm (100 mW, LBX-488-100-CSB-OE, Oxxius) was directed through the back-port of an inverted microscope (Nikon Te2000-U). A dichroic mirror (DI03-R405/488/561/635, Semrock) reflected the beam through an oil-immersion objective lens (Nikon CFI Plan Apochromat VC 100x Oil, NA 1.4, W.D 0.13 mm) which focused on a diffraction-limited confocal spot. The emitted fluorescence was collected by the objective lens and was passed through the same

dichroic before being focused by the tube lens through a 50 µm pinhole (Thorlabs). The AF488 and AF594 fluorescence were separated by a second dichroic filter (LP02-647RU-25, Semrock). The AF594 fluorescence was passed through a band-pass filter (FF01-629/53, Semrock) before being focused onto an Avalanche Photodiode Detector (APD) (PerkinElmer). The AF488 fluorescence passed through a second filter set (long-pass: BLP01-488R-25 and band-pass: FF01-525/30-25, Semrock) onto a second APD. Outputs from the APDs were connected to a USB data acquisition card (USB-CTR04, Measurement Computing), which counts the signals and combines them into time-bins of 50 µs, the expected residence time of the molecules in the confocal volume. The data were analyzed as in [15] using custom-written scripts written in Igor Pro (Wavemetrics). Coincident events were those that had at least 10 photon counts bin^{-1} in each channel. After accounting for autofluorescence and crosstalk (Equation 1-3), the FRET efficiency (Equation 1) and approximate size of each oligomer (Equation 4) was calculated from the intensities in each channel:

Approximate size = 2ID+1IAImonomer Equation 4

where γ is the experimentally determined gamma factor, corresponding to the relative detection efficiencies of the two dyes by the instrumentation and their quantum yields, and $I_{monomer}$ is the average monomer brightness, calculated from the average of the non-coincident donor channel bursts intensities.

Oligomers were divided into three groups depending on their approximate size as already published [15, 16]. Small oligomers correspond to apparent dimers, and the remaining oligomers are classified as medium in size (up to 20 monomer units/oligomer). The division into type-A and type-B oligomers was made by globally fitting the medium-sized oligomer FRET efficiency histograms (shared x-center and x-width for all time-points/treatments) to two Gaussian distributions (type-A have a lower FRET efficiency than type-B). The global fitting ensures that the same populations are identified from each dataset. To determine the effect of the fitting error on the overall calculation of the populations, the errors were propagated for one dataset and are represented in Extended Data Fig.2d & 3a.

The fraction of coincidence was calculated by dividing the number of coincident events by the total number of events having counts above 10 photons bin^{-1} in the AF488 channel. For comparison, a dual-labelled DNA sample in which ~100% of the molecules should be able to undergo FRET, the coincidence is ~0.10 under these experimental conditions [17].

To assess the systematic error of utilizing FRET efficiency to measure proximity, we determined our ability to separate two populations exhibiting different proximity ratios. To do this, we made use of labelled DNA oligonucleotides with a donor/acceptor FRET dye pair separated by two different distances (Duplex-1 formed from ODN-1 and ODN-2, and Duplex-2 formed from ODN-1 and ODN-3)[2], exhibiting similar proximity ratios to those shown by the oligomers (Supplementary Table 2 for Oligonucleotide sequences).

Duplex-1 and Duplex-2 were mixed together at different ratios (1:1, 1:3, and 3:1), diluted to a final total concentration of 25 pM and measured on the single-molecule confocal microscope using the same experimental conditions used for analysing the cell lysate samples. The proximity ratios were determined using Equations 1-3, and FRET efficiency histograms were plotted and fit to two Gaussian distributions (Extended Data Fig2 ei & ii.),

which were integrated and normalised to the total number of donor bursts, as was done to determine the populations of type-A and type-B oligomers. The DNA duplexes would be equivalent to protein dimers (i.e. two dyes present). Due to the uncertainty in distinguishing different FRET efficiencies in the dimer-like population, we treat this population separately. For larger oligomers with many more dyes, we can confidently separate the type A and type B populations.

Proteinase-K digestion

Susceptibility to Proteinase-K was measured to confirm the presence or absence of distinct oligomer populations (Type-A and B) for both AF488- and AF594-labeled recombinant A53T and cell lysate A53T α-Syn oligomer samples. Aggregate samples were diluted 1 in 500 into a range of concentrations of Proteinase-K (0, 0.001, 0.01, 0.1, 0.1, 1, 4, 10 μg/ml) in 25 mM Tris-HCl (pH 7.4), 100 mM NaCl, 1 mM $CaCl_2$ with incubation for 10 minutes at 37 ^{o}C. Samples were then diluted 1 in 500 into 25 mM Tris-HCl (pH 7.4), 100 mM NaCl to stop the enzyme reaction and were analyzed using single-molecule confocal microscopy.

Imaging aggregates in cell lysates

To determine endogenous aggregates in SNCA-A53T cells, cell lysates from hiPSC derived neurons (day 70) of SNCA-A53T or iso-CTRL were collected by mechanically breaking the pellets followed by spinning them down at 10,000 rpm for 10 min. The cell lysates were stored at -80 °C until use. The lysates were diluted 10-fold into PBS before being incubated with 100 nM of the aggregate-specific aptamer (Apt-DS, Table below, ATDBio) for 15 minutes. Borosilicate glass coverslips (20 × 20 mm, VWR International, USA. Product number 63 1-0122) were cleaned using an argon plasma cleaner (Zepto, Diener, Germany) for 40 minutes to remove any fluorescent residues. Frame-Seal slide chambers (9 × 9 mm^2, Biorad, Hercules, USA. Product number SLF-0601) were affixed to the glass, and the lysate and aptamer were added to the cover slip on the inside of the chamber and incubated for at least 30 minutes. The coverslip was subsequently washed 3x with PBS, and a solution containing 100 nM Apt-DS and 5 nM imaging strand (IS, Supplementary Table3, ATDBio).

Imaging was performed using on the TIRF microscope described elsewhere in the methods using ~50 W cm^{-2} 638 nm laser irradiation. The exposure time was 50 ms and 8000 frames were recorded. The positions of the transiently immobilized imaging strands within each frame were determined using the PeakFit plugin (an imageJ/Fiji plugin of the GDSC Single Molecule Light Microscopy package (http://www.sussex.ac.uk/gdsc/intranet/microscopy/imagej/gdsc_plugins) for imageJ using a signal strength threshold of 30 and a precision threshold of 20 nm. The localizations were sorted into clusters using the DBSCAN algorithm in Python 3.9 (sklearn v0.24.2) using epsilon = 1 pixels and a minimum points threshold of 40 to remove spurious localizations. Each cluster was defined as an aggregate and the number per field of view was determined.

Sample preparation for single-molecule localization microscopy

For single-molecule localization microscopy (SMLM), iPSC-derived cortical neurons were grown on glass coated Ibidi chambers. The neurons were washed once in PBS, followed by a 15-minute fixation in 4% paraformaldehyde + 0.1% glutaraldehyde (both from Electron Microscopy Services) in PBS at RT, followed by a 7-minute reduction in 0.1% sodium borohydride (Sigma) in at RT. Neurons were then washed 2x in PBS.

The neurons were permeabilized with 0.25% triton X-100 in PBS for 10 minutes at RT followed by non-specific blocking in blocking solution (0.1% triton X-100, 10% normal goat serum (Abcam), 10% salmon sperm DNA (Thermo Fisher Scientific)) in PBS for 2 hours at RT. The samples were then incubated with 100 nM of the aptamer (sequence: GCCTGTGGTGTTGGGGCGGGTGCGTTATACATCTA) made up in the blocking solution at 4°C overnight. After incubation, neurons were washed 1x in PBS and incubated with a primary Tom20 antibody (Santa-cruz sc-17764) 1:100 made up in the blocking solution for 1 hour at RT. Neurons were then washed 1x in PBS and incubated with a secondary anti-mouse conjugated Alexa Fluor 647 (abcam) made up in blocking solution for 1 hour at RT, followed by two PBS washes before imaging.

Single-molecule localization microscopy in cells

For single-molecule localization microscopy (SMLM), iPSC-derived cortical neurons were grown on glass coated Ibidi chambers. The neurons were washed once in PBS, followed by a 15-minute fixation in 4% paraformaldehyde + 0.1% glutaraldehyde (both from Electron Microscopy Services) in PBS at RT, followed by a 7-minute reduction in 0.1% sodium borohydride (Sigma) in at RT. Neurons were then washed 2x in PBS.

The neurons were permeabilized with 0.25% triton X-100 in PBS for 10 minutes at RT followed by non-specific blocking in blocking solution (0.1% triton X-100, 10% normal goat serum (Abcam), 10% salmon sperm DNA (Thermo Fisher Scientific)) in PBS for 2 hours at RT. The samples were then incubated with 100 nM of the aptamer (sequence: GCCTGTGGTGTTGGGGCGGGTGCGTTATACATCTA) made up in the blocking solution at 4°C overnight. After incubation, neurons were washed 1x in PBS and incubated with a primary Tom20 antibody (Santa-cruz sc-17764) 1:100 made up in the blocking solution for 1 hour at RT. Neurons were then washed 1x in PBS and incubated with a secondary anti-mouse conjugated Alexa Fluor 647 (abcam) made up in blocking solution for 1 hour at RT, followed by two PBS washes before imaging.

SMLM was performed on a Nanoimager super-resolution microscope (Oxford Nanoimaging Ltd) equipped with an Olympus 1.4 NA 100x oil immersion super apochromatic objective. To ensure efficient blinking for STORM (AF647-tagged tom20), the samples were incubated with a blinking induction buffer (B cubed, ONI). Separately, DNA-PAINT was also employed using an imaging strand (sequence: CCAGATGTAT-CY3B) which recognizes the end sequence of the aptamer. 1 nM of the imaging strand was added to the B cubed buffer before imaging. The laser illumination angle was set to 51° for all imaging leading to TIRF. AF647-tagged tom20 was first imaged for 10000 frames using the 640 nm laser (80% power). After this, 4000-5000 frames at 30% power for the 561 nm laser was used to image and super-resolve the aptamer.

Both were recorded at a frame-rate of 50 ms. This was done for 2-3 fields of view per line and condition.

SMLM performed on the Nanoimager was partly analyzed using the Oxford Nanoimaging Ltd developed online software, CODI. Initially, an inbuilt drift-correction was performed to correct single-molecule localizations in case the sample drifted during acquisition. After the drift correction, the number of frames was changed to only include the frames where the relevant fluorophore was imaged. Each localization was fitted to a 2D Gaussian distribution and any of those with a standard deviation larger than 250 nm were removed. Finally, any localizations with an error lower than 20 nm were discarded. Once the filtered super-resolved image was generated, density-based spatial clustering of applications with noise (DBSCAN) was performed on the resulting images. Each cluster in DBSCAN needed to have at least 15 localizations, and each localization had to be within 60 nm of each other. This was to remove any non-specific aptamer binding, and to only detect aggregates that were quite spatially confined.

MitoGFP transfection

iPSC derived neurons were transduced using a mito-GFP expressing lentivirus. The pLYS1-FLAG-MitoGFP-HA plasmid (#50057, Addgene) was packaged using a third-generation lentiviral system, with packaging plasmids pRSV-Rev (#12253, Addgene) and pMDLg/pRRE (#12251, Addgene) and envelope plasmid pVSV-G (#138479, Addgene). Constructs were incubated at a 2:1:1:2 ratio with pRSV-Rev (30 g), pMDLg/pRRE (15 g), pVSV-G (15 g) and plasmid DNA (30 g), and 72.4 of Lipofectamine™ 2000 Transfection Reagent in 8 mL Opti-MEM™ Reduced Serum Medium, no phenol red. The mix was incubated at RT for 20 minutes before being slowly added to HEK 293FT cultures. The following morning, the media was aspirated and replaced with 25 mL of DMEM, high glucose, GlutaMAX™ Supplement supplemented with 10% foetal bovine serum (FBS; 12103C; Sigma Aldrich) and 1% Penicillin-Streptomycin. The HEK 293FT cells were incubated for 48 hours at 37 °C and 5% CO_2. After 48 hours, the media was collected and centrifuged at 1500 rpm for 5 minutes to remove cell debris. The supernatant was filtered through a 0.45 m Minisart™ syringe filter Lentiviruses were then concentrated by ultracentrifugation of the cell supernatant at 48,000 rpm for three hours at 4 °C. Viral pellets were resuspended in ice cold PBS, aliquoted, snap-frozen and stored at -80 °C. Cells were treated with the virus at a dilution of 1:100 overnight, and the media changed the following morning.

RNA extraction, cDNA synthesis, and quantitative polymerase chain reaction (qPCR):

Cell pellets were snap-frozen using dry ice. RNA was harvested using the Maxwell® RSC simplyRNA Cells kit (Promega), and the Maxwell® RSC 48 instrument. After RNA extraction, RNA concentration and quality were assessed using a nanodrop. Up to 2 µg of RNA was reverse-transcribed into cDNA using the High-Capacity cDNA Reverse Transcription kit (ThermoFisherScientific). qPCR was performed using PowerUp SYBR Green Master Mix (Applied Biosystems) according to the manufacturer's instructions. Samples were run for each

gene in technical triplicate on the QuantStudio 6 Flex Real-Time PCR System (Applied Biosystems). A reverse transcriptase-negative control (-RT) and no-template control (NTC) were included as negative controls. SNCA expression levels were normalized to the housekeeping gene, GAPDH, following the delta-delta Ct method. Primers were as follows: *SNCA*, forward AAGAGGGTGTTCTCTATGTAGGC and reverse GCTCCTCCAACATTTGTCACTT. *GAPDH*, forward ATGACATCAAGAAGGTGGTG and reverse CATACCAGGAAATGAGCTTG.

Single vesicle-based membrane permeabilization assay

For the membrane permeabilization assay, vesicles were prepared as previously described [18]. Using this assay, it has been previously shown that α-Syn oligomers disrupt and permeabilize membranes [19, 20]. Briefly, vesicles were synthesized using Phospholipids 16:0-18:1 PC and biotinylated lipids 18:1-12:0 Biotin PC using freeze thaw method with mean diameter of 200 nm. Each vesicle was filled with 100 µM Cal-520 dye and immobilized in PLL-g-PEG coated plasma cleaned glass coverslips using biotin-neutravidin linkage. The surroundings of the vesicles were filled with Ca^{2+} buffers. 50 µL of the sample was incubated with the vesicles for 15 minutes, and Ca^{2+} influx was quantitatively measured using a homebuilt Total Internal Reflection Fluorescence Microscope (TIRFM) based on an inverted Nikon Ti microscope. The 488 nm laser was a focused back-focal plane of the 60X, 1.49NA oil immersion objective lens used to excite the Cal-520 dye. The fluorescence signal was collected by the same objective and passed through an appropriate set of filters before imaged in an EMCCD camera.

ELISA assay

To determine the concentrations of α-Syn monomers or oligomers, WT or A53T unlabeled monomer-treated and untreated cell lysates were collected at various time points. The collected lysates were stored at -80 °C. Oligomeric α-Syn was analyzed using the Human Synuclein, alpha (non A4 component of amyloid precursor) oligomer (SNCA oligomer) ELISA kit (CSB-E18033h, Generon) and monomeric α-Syn was analyzed using the LEGEND MAX™ Human α-Synuclein ELISA Kit (SIG-38974, BioLegend) according to the manufacturer's instructions. The levels of monomeric and oligomeric α-Syn were normalized to the total protein concentration, as determined by the Pierce BCA Protein Assay Kit (23225, Thermo Fisher Scientific).

Correlative Light and Transmission Electron Microscopy (CLEM)

Cells were plated and grown in 35 mm gridded glass-bottom dishes (P35G-1.5-14-CGRD, Mattek). At the appropriate time point, an equal volume of fixative (8% Formaldehyde in 0.2 M phosphate buffer (PB), pH 7.4), pre-warmed to 37 °C, was added directly to the growth medium and left for 15 mins. Cells were then washed in 0.1 M PB, and confocal images using Zeiss LSM 880 (63x/1.4 oil DIC UV-VIS-IR M27 objective) then acquired from the area of interest (LD LCI Plan-Apochromat 25x/0.8 Imm Korr DIC M27 objective for tiling scan).

Samples were then post-fixed in 2.5% Glutaraldehyde/ 4% Formaldehyde in 0.1 M PB (pH 7.4) for 30 mins, stained in 1% reduced osmium (1% osmium/ 1.5% potassium ferricyanide) at 4 °C for one hour, followed by 1% tannic acid for 45 mins at RT, prior to quenching in 1% sodium sulfate at RT for 5 mins and washing in double-distilled water (3 x 5 mins). The glass coverslip was then removed from the Mattek dish, and dehydrated through a graded series of ethanol (25%, 50%, 70%, 90% and 100% for 5 mins each), embedded in Durcupan (44610-1EA, Sigma-Aldrich) and polymerised at 65 °C for 48 hrs. The glass coverslip was removed from the resin by submerging in liquid nitrogen, and the cells of interest relocated using the alphanumeric grid imprinted on the surface of the resin block. The block was trimmed to the cell of interest, serial sectioned at a thickness of 70 nm using a UC7 ultramicrotome (Leica Microsystems) and a diamond knife (Diatome) and collected onto 2 x 1 mm copper slot grids with a formvar support film (G089, TAAB Laboratories Equipment). Sections were post-stained using Reynold's lead citrate and 1% uranyl acetate. Serial images of the cell were then acquired using a Transmission Electron Microscope (TEM; Tecnai Spirit BioTwin; ThermoFischer Scientific) operated at 120 keV.

Serial TEM images were aligned using the TrakEM plugin in FIJI [65] (www.fiji.sc) and aligned to the confocal images using BigWarp [25] (www.fiji.sc).

Correlative light and focused ion beam scanning electron microscopy

Cells were cultured, fixed and imaged using confocal microscopy as described above. For post-fixation and staining, a modified version of the NCMIR method was used [26]. Cells were post-fixed in 2.5% glutaraldehyde/ 4% formaldehyde in 0.1 M PB (pH 7.4) at RT for 30 mins, and stained in 1% reduced osmium (1% osmium/ 1.5% potassium ferricyanide) at 4 °C for 1 hr. Cells were then treated with 1% thiocarbohydrazide (TCH) for 20 mins at RT, followed by 2% osmium tetroxide for 30 mins at RT, and incubated overnight at 4 °C in 1% uranyl acetate. The following day, cells were stained *en bloc* with lead aspartate (pH 5.5) at 60 °C for 30 mins. The glass coverslip was then removed from the Mattek dish and underwent graded dehydration in ethanol (25%, 50%, 70%, 90% and 100%, 5 mins per step) followed by embedding in Durcupan and polymerization at 65 °C for 48 hours.

The coverslips were subsequently removed from the resin block using liquid nitrogen, and the cells relocated using grid coordinates. Areas of 3 x 3 grid squares in size with the cell of interest located at the center were then trimmed out using a razor blade, removed from the resin block [21] and mounted on a 12.7 mm SEM pin stub (10-002012-100, Labtech) using silver paint (AGG3691, Agar Scientific). After mounting, each sample was sputter coated with a 10 nm layer of platinum (Q150S, Quorum Technologies).

Focused ion beam scanning electron microscopy (FIB SEM) was carried out using a Crossbeam 540 FIB SEM with Atlas 5 for 3-dimensional tomography acquisition (Zeiss, Cambridge). The cells of interest were relocated by briefly imaging through the platinum coating at an accelerating voltage of 20 kV with 500 pA current, and correlating to the previously acquired confocal images. Once relocated, reoriented, and prepared for serial imaging, electron micrographs of the region of interest were acquired at 5 nm isotropic resolution, using a 11 µs dwell time. During acquisition, the SEM was operated at an accelerating voltage of 1.5 kV

with 1 nA current. The EsB detector was used with a grid voltage of 1,200 V. Ion beam milling was performed at an accelerating voltage of 30 kV and current of 700 pA.

After cropping to the region of interest using Fiji, the datasets comprised 2,337 slices with an approximate volume of 1,695 μm^3 (21.3 μ m x 6.8 μ m x 11.7 μ m) for sample 51, and 2,001 slices with an approximate volume of 1,428 μm^3 (17.2 μ m x 8.3 μm x 10 μm) for sample 52. Each stack was registered using the Atlas 5 autotune marks (template matching by normalized cross-correlation; https://sites.google.com/site/qingzongtseng/template-matching-ij-plugin) and batch processed to suppress noise and enhance sharpness and contrast (i. Gaussian blur 0.8 pixel radius; ii. smart sharpening with highlights suppressed: radius 10 pixels, strength 60%, then radius 1.2 pixels, strength 150%; iii. 8-bit grayscale conversion; Adobe Photoshop 2020). For reorientation in the image plane of the matching confocal datasets, each image stack was rotated and resliced perpendicular to the original coverslip plane, prior to alignment with the confocal dataset using BigWarp [24].

CLEM: Generation of error maps

Error maps were created using the eC-CLEM plugin [22] in Icy [23] using the following workflow. The landmarks points used in the initial BigWarp registration were exported as a .csv file. The file was then reformatted to be imported into eC-CLEM. This involved deleting the first 2 columns of the .csv file and splitting the file into 2, one for the coordinate of the points in the light microscopy (LM) data, one for the electron microscopy (EM) data. The LM and EM image stacks were then opened in Icy and eC-CLEM started. The landmark points were added to each dataset using the 'Import Roi from csv file (Amira or other)' plugin found in eC-CLEM in 'Advanced usage'. The LM and EM data were then aligned using the '3D but let me update myself' mode. In this mode, a rigid transformation (rotation, translation and scaling) is applied to the light microscopy data. Once the transformation was calculated, an error map was calculated, indicating the average registration error in any point of the transformed LM image. The color bar reflects the registration error value in nanometers expected in each pixel of the LM image.

Liposome preparation

Cardiolipin, 1,2-dimyristoyl-sn-glycero-3-phosphocholine (DMPC) (Avanti polar lipids) and biotinylated cardiolipin (Echelon Biosciences) were obtained as lyophilized powders. About 1.5 mg of each lipid was transferred into glass test tubes and dissolved in 2 ml of chloroform: methanol (3:1) solvent. A thin layer of lipid was prepared by continuously rotating the test tube while treating the sample with a gentle stream of N_2 gas until all the solvent evaporated. Test tubes were kept under vacuum overnight to remove any trace amount of solvent. The dry lipid layers were rehydrated with an appropriate amount of 25 mM Tris buffer (pH 7.4) to make a final concentration of 2 mM and vortexed for 2 mins to dissolve the lipid layer into the buffer. Biotinylated cardiolipin liposomes were prepared in a similar way by adding 2% (M/M) biotin-conjugated cardiolipin before processing for liposomes preparation. Mixed vesicles followed an extra step of incubation at 40 °C with constant stirring to allow for

homogeneous distribution of lipids in the liposomes. Small unilamellar vesicles were prepared by bath sonication (VWR ultrasonic) until a clear solution was obtained, indicating formation of unilamellar vesicles. Lipid vesicles were stored at 4 °C in glass vials until further use.

Cardiolipin exhibits polymorphic phase behavior in the aqueous medium, which depends upon the salt concentration. In the absence of, or in low salt concentrations, cardiolipin adopts a micellar form, whereas, in the presence of high salt concentration (1-2 M), it transforms into an inverted hexagonal structure [24](reviewed here [25]). Since in our work, we performed the aggregation assay in 25 mM Tris and in the absence of salt, CL would favor the micellar form under these conditions. Moreover, our CD data shows a change of secondary structure of A53T from the random coil in the solution to α-helical form in the presence of liposomes, which confirms that the headgroup of lipids are oriented outward and available to bind protein molecules.

TIRFM: cardiolipin imaging.

Slide preparation

Biotinylated liposomes were prepared as described previously [17]. Briefly, glass coverslips (22 × 22 mm) were cleaned via incubation in an argon plasma oven for 1h prior to use. Plasma cleaned coverslips were affixed to Frame-Seal slide chambers (9 × 9 mm^2, Biorad, Hercules, CA), and the chamber was filled with about 50 µl poly-L-lysine solution (Sigma Aldrich) and incubated for 30 minutes at RTin a covered box. Glass coverslips were washed twice with filtered Tris buffer, after which a sample containing α-Syn fibrils prepared in the presence of cardiolipin was added in the chamber, and the coverslip was placed on the microscope stage for imaging.

TIRFM imaging

Imaging was performed in a home built total internal reflection microscope as described previously (Janeczek et al., 2017). Amyloid fibrils prepared in the presence of biotin-conjugated cardiolipin were incubated with 1 nM Alexa Fluor 647 (AF647)-streptavidin (Thermo Fisher Scientific) and 5 mM ThT (Sigma Aldrich) for 10 minutes at RT. Images were recorded for 50 frames from the red channel (AF647 emission) with 641 nm illumination, followed by the green channel (ThT emission) with 488 nm illumination.

Data analysis

The images generated from (AF647)-streptavidin and SAVE imaging with ThT were analyzed using ImageJ and a custom written script in Igor Pro (Wavemetrics). Each image stack was first averaged over the 50 frames before the background was subtracted using ImageJ's *Subtract Background* feature with a rolling ball radius of 50 pixels. The images from each channel were loaded into Igor Pro, and a threshold of 4000 ADUs was applied in the SAVE image to identify

pixels in which aggregates were present. The individual aggregates were segmented and were defined as being coincident if any of the corresponding AF647 channel pixels had intensity values greater than 4000 ADUs. The percentage coincidence is determined from the number of coincident aggregates and the total number of aggregates detected. The mean and standard deviations are calculated across the image sets.

Single vesicle-based membrane permeabilization assay

For the membrane permeabilization assay, vesicles were prepared as previously described [18]. 50 µL of the sample was incubated with the vesicles for 15 minutes, and Ca^{2+} influx was quantitatively measured using a homebuilt Total Internal Reflection Fluorescence Microscope (TIRFM) based on an inverted Nikon Ti microscope.

Immunohistochemistry

Cells were fixed in 4 % paraformaldehyde and permeabilized with 0.2 % Triton-X 100. 5 % BSA was used to block non-specific binding before cells were incubated with primary antibodies either for 2 hours at room temperature or overnight at 4 °C. The next day, cells were washed three times with PBS and incubated with secondary antibody for 1hr at room temperature. Cells were mounted with antifading medium after three times wash steps (DAPI was added in the second wash if required) and let dry overnight.

Aptamer staining

For ATTO 425 labelled Aptamer staining, cells were permeabilized with 0.25 % Triton X-100 and blocked with 10 % normal goat serum (NGS) for 20 min followed by another 3 hours with 0.1 % Trion X-100 and 10 % NGS. Then cells were overnight incubated with 0.5uM Aptamer. After washing three times with PBS, cells were mounted with antifading medium [26].

SI Method references

1. Hoyer, W., et al., *Dependence of alpha-synuclein aggregate morphology on solution conditions.* J Mol Biol, 2002. **322**(2): p. 383-93.
2. Thirunavukkuarasu, S., E.A. Jares-Erijman, and T.M. Jovin, *Multiparametric fluorescence detection of early stages in the amyloid protein aggregation of pyrene-labeled alpha-synuclein.* J Mol Biol, 2008. **378**(5): p. 1064-73.
3. Cremades, N., et al., *Direct observation of the interconversion of normal and toxic forms of alpha-synuclein.* Cell, 2012. **149**(5): p. 1048-59.
4. Maltsev, A.S., J. Ying, and A. Bax, *Impact of N-terminal acetylation of α-synuclein on its random coil and lipid binding properties.* Biochemistry, 2012. **51**(25): p. 5004-13.

5. Kang, L., et al., *Mechanistic insight into the relationship between N-terminal acetylation of α-synuclein and fibril formation rates by NMR and fluorescence.* PLoS One, 2013. **8**(9): p. e75018.
6. Kang, L., et al., *N-terminal acetylation of α-synuclein induces increased transient helical propensity and decreased aggregation rates in the intrinsically disordered monomer.* Protein Sci, 2012. **21**(7): p. 911-7.
7. Lima, V.A., et al., *Role of Parkinson's Disease-Linked Mutations and N-Terminal Acetylation on the Oligomerization of α-Synuclein Induced by 3,4-Dihydroxyphenylacetaldehyde.* ACS Chem Neurosci, 2019. **10**(1): p. 690-703.
8. Chen, Y., et al., *Engineering synucleinopathy-resistant human dopaminergic neurons by CRISPR-mediated deletion of the SNCA gene.* Eur J Neurosci, 2019. **49**(4): p. 510-524.
9. Bartolomé, F. and A.Y. Abramov, *Measurement of mitochondrial NADH and FAD autofluorescence in live cells.* Methods Mol Biol, 2015. **1264**: p. 263-70.
10. Esteras, N., et al., *Mitochondrial hyperpolarization in iPSC-derived neurons from patients of FTDP-17 with 10+16 MAPT mutation leads to oxidative stress and neurodegeneration.* Redox Biol, 2017. **12**: p. 410-422.
11. Gandhi, S., et al., *PINK1-associated Parkinson's disease is caused by neuronal vulnerability to calcium-induced cell death.* Mol Cell, 2009. **33**(5): p. 627-38.
12. Zamaraeva, M.V., et al., *Ionophoretic properties of ferutinin.* Cell Calcium, 1997. **22**(4): p. 235-41.
13. Abramov, A.Y. and M.R. Duchen, *Measurements of threshold of mitochondrial permeability transition pore opening in intact and permeabilized cells by flash photolysis of caged calcium.* Methods Mol Biol, 2011. **793**: p. 299-309.
14. Maftah, A., J.M. Petit, and R. Julien, *Specific interaction of the new fluorescent dye 10-N-nonyl acridine orange with inner mitochondrial membrane. A lipid-mediated inhibition of oxidative phosphorylation.* FEBS Lett, 1990. **260**(2): p. 236-40.
15. Horrocks, M.H., et al., *Fast flow microfluidics and single-molecule fluorescence for the rapid characterization of α-synuclein oligomers.* Anal Chem, 2015. **87**(17): p. 8818-26.
16. Tosatto, L., et al., *Single-molecule FRET studies on alpha-synuclein oligomerization of Parkinson's disease genetically related mutants.* Sci Rep, 2015. **5**: p. 16696.
17. Horrocks, M.H., et al., *Single-Molecule Imaging of Individual Amyloid Protein Aggregates in Human Biofluids.* ACS Chem Neurosci, 2016. **7**(3): p. 399-406.
18. Flagmeier, P., et al., *Ultrasensitive Measurement of Ca(2+) Influx into Lipid Vesicles Induced by Protein Aggregates.* Angew Chem Int Ed Engl, 2017. **56**(27): p. 7750-7754.
19. Lee, J.-E., et al., *Mapping Surface Hydrophobicity of α-Synuclein Oligomers at the Nanoscale.* Nano letters, 2018. **18**(12): p. 7494-7501.
20. Varela, J.A., et al., *Optical Structural Analysis of Individual alpha-Synuclein Oligomers.* Angew Chem Int Ed Engl, 2018. **57**(18): p. 4886-4890.
21. Russell, M.R., et al., *3D correlative light and electron microscopy of cultured cells using serial blockface scanning electron microscopy.* J Cell Sci, 2017. **130**(1): p. 278-291.
22. Paul-Gilloteaux, P., et al., *eC-CLEM: flexible multidimensional registration software for correlative microscopies.* Nat Methods, 2017. **14**(2): p. 102-103.
23. de Chaumont, F., et al., *Icy: an open bioimage informatics platform for extended reproducible research.* Nat Methods, 2012. **9**(7): p. 690-6.

24. Sankaram, M.B., G.L. Powell, and D. Marsh, *Effect of acyl chain composition on salt-induced lamellar to inverted hexagonal phase transitions in cardiolipin.* Biochim Biophys Acta, 1989. **980**(3): p. 389-92.

25. Dahlberg, M., *Polymorphic phase behavior of cardiolipin derivatives studied by coarse-grained molecular dynamics.* J Phys Chem B, 2007. **111**(25): p. 7194-200.

26. Whiten, D.R., et al., *Nanoscopic Characterisation of Individual Endogenous Protein Aggregates in Human Neuronal Cells.* Chembiochem, 2018. **19**(19): p. 2033-2038.

25. Bogovic, J.A., Hanslovsky, P., Wong, A., and Saalfeld, a. (2016) Robust registration of calcium images by learned contrast synthesis, ISBI, 2016 1123-1126.

26. Deerinck, T.J., Bushong, E.A., Lev-Ram, V., Shu, X., Tsien, R.Y., and Ellisman M.H. (2010)

Enhancing Serial Block-Face Scanning Electron Microscopy to Enable High Resolution 3-D. Microsc. Microanal, 2010 vol. 16 1138-1139.

Code	Sequence
ODN-1	AF488-AAATCTAAAGTAACATAAGGTAACATAACGGTAAGTCCA
ODN-2	TGGACTTACCGTTATGTTACCTTATGTTACTT6AGATTTA
ODN-3	TGGACTTACCGTTATGTTACCT6ATGTTACTTTAGATTTA

Supplementary Table 2. Oligonucleotide sequences. 6 = amino-C6-dT labelled with Alexa Fluor 647.

**Oligonucleotide**	**Sequence**
Apt-DS	GCCTGTGGTGTTGGGGCGGGTGCGTTACCACCACCACCACCACCA
IS	GGTGGT-ATTO 655

Supplementary Table 3. Oligonucleotide sequences for Apt-DS and IS.

Antibody Type	Antibody name	Supplier name	Cat No.	Dilution
Primary	Anti-MAP2	abcam	ab183830	1 : 500
	Anti-TRB1	abcam	ab31940	1 : 500
	Anti-Alpha-synuclein antibody [MJFR1]	abcam	ab138501	1 : 250
	Anti-Alpha-synuclein aggregate antibody [MJFR-14-6-4-2]	abcam	ab209538	1 : 200
	TOMM20	Santa Cruz	sc-17764	1 : 100
Secondary	Goat Anti-Chicken IgY H&L (Alexa Fluor® 488)	abcam	ab150169	1 : 500
	Goat Anti-Mouse IgG H&L (Alexa Fluor® 555)	abcam	ab150114	1 : 500
	Goat Anti-Rabbit IgG H&L (Alexa Fluor® 647)	abcam	ab150079	1 : 500

Supplementary Table 5. Details of the antibodies used for this study.
